# Social and political trust diverge during a crisis

**DOI:** 10.1038/s41598-023-50898-4

**Published:** 2024-01-03

**Authors:** Arnstein Aassve, Tommaso Capezzone, Nicolo’ Cavalli, Pierluigi Conzo, Chen Peng

**Affiliations:** 1 DONDENA Center for Research on Social Dynamics and Public Policy, Bocconi University, Milan, Italy; 2 Collegio Carlo Alberto, Turin, Italy; 3 Department Economics and Statistics “Cognetti de Martiis”, University of Turin, Turin, Italy

**Keywords:** Human behaviour, Psychology and behaviour

## Abstract

This study shows that social and political trust may diverge in the face of shared threats, and that this pattern is driven by negative information about crisis management. Leveraging a three-wave panel survey and an information-provision experiment in the USA during the COVID-19 crisis, our research reveals that negative perceptions of pandemic management lead to a decline in political trust and a parallel increase in social trust. This dynamic is pronounced among government supporters, who, confronted with COVID-19 challenges, experience a substantial erosion of political trust. Simultaneously, there is a notable rise in social trust within this group. Our analysis suggests that, as government supporters attributed more responsibility for the crisis to their political leader, political trust was supplanted by social trust. Disenchanted voters, feeling let down by institutions, sought support in society. Both the survey and the experiment underscore that societal shocks can prompt individuals to shift from relying on formal institutions to informal ones as a coping strategy. This research contributes a generalizable framework explaining how negative perceptions of crisis management can lead societies to substitute political trust with social trust, advancing our understanding of societal responses to shared threats and adaptive strategies during crises.

## Introduction

Other than the obvious health risks, the COVID-19 pandemic brought out anxiety about current and prospective jobs^1,2^ and limitations on social interactions^3–5^. More generally, it influenced our way of dealing with strangers^6–8^ and attitudes towards institutions^7,9,10^. With a combination of panel-data analysis and experimental techniques, this study investigates shifts in social and political trust in response to varying perceptions of governmental pandemic management, offering fresh insights into a widely discussed topic.

The extant literature offers competing hypotheses but focuses on social and institutional trust separately. On the one hand, political trust may vary depending on the perceived performance of politicians^11,12^, and on whether citizens “rally round the flag”^13^. Unexpected events, on the other hand, may influence social trust too. While some assert that crises should not alter social trust^14,15^, other studies suggest the opposite^16–20^, without a consensus on the direction of change. The most recent literature on the pandemic’s impact on social and political trust, both separately^8,21–24^ and jointly^7,25,26^, tends to focus on a general “rally” effect emerging at the onset of the pandemic. However, it largely overlooks the role of the perceived performance of ruling political forces in shaping individuals’ attitudes during the later stages of the crisis.

Examining the dynamic interaction between social and political trust, this study introduces novel evidence, revealing a potential divergence between social and political trust during a shared, unforeseen threat. Crucially, the research delves into a specific mechanism fueling this divergence: citizens’ disappointment with the management of the crisis by political institutions.

This paper makes a fourfold contribution. Firstly, it provides unique survey evidence using longitudinal data to simultaneously explore social and political trust, observing how pandemic experiences and policy changes correlate with citizens’ attitudes over time and across locations. Secondly, by tracking the evolution of political trust, it tests the persistence of the rally effect, showcasing adjustments in citizens’ trust levels in response to crisis management evaluations. Thirdly, it investigates variations in these dynamics based on respondents’ political orientation and their attribution of responsibility for the pandemic. Fourthly, the paper integrates survey-based observational data with an information-provision experiment, addressing issues of unobserved heterogeneity and reverse causality to assess the persistence of hypothesized mechanisms in the longitudinal analysis, particularly regarding the perceived (mis)management of the crisis.

We administered a longitudinal survey in the United States (US) at three key time points: before the peak of the initial pandemic wave, immediately after that peak, and at the onset of the second pandemic wave just prior to the 2020 federal elections. Subjects were asked standard questions on social trust and trust towards different institutional targets and on the attribution of responsibility for managing the pandemic crisis. In the third survey wave, we implemented an experiment where participants were exposed to either positive or negative information about the country’s health and economic performance, and then elicited their levels of social and political trust. In the first survey wave, we gathered data on respondents’ voting behavior in the previous presidential elections, enabling us to investigate whether social and political trust exhibited partisan trends.

Our descriptive findings reveal a temporal shift in trust dynamics, with an overall decrease in political trust and a slight increase in social trust when the first pandemic wave peaked. These trends, however, exhibit distinct patterns based on political affiliation. The decline in political trust is primarily attributed to Republicans, who witnessed a decline in trust in their political leader (Trump) and the federal government. This decline appears to be a consequence of the escalating incidence and mortality associated with the pandemic, which casted doubts on the effective management of the crisis. Consistent with this interpretation, this decline was most pronounced during the transition from the first to the second pandemic wave, indicating a heightened political disillusionment over that period. Conversely, while social trust among Democrats remained relatively stable, Republicans exhibited a slight increase in their propensity to trust strangers.

These partisan variations are amplified when considering personal exposure to COVID-19. Furthermore, the attribution of responsibility for crisis management followed a pattern similar to the trend in political trust, underscoring a surge in disappointment, especially among Republicans, regarding the political institutions they expected support from. Overall, these descriptive findings underscore a divergence between social and political trust stemming from disillusionment with the handling of the crisis. This phenomenon is more clearly identified through our information-provision experiment, which documents a consistent dynamic in social and political trust among Trump voters. When exposed to negative information regarding the U.S.’s COVID-19 management, Trump voters marginally increased their social trust and concurrently decreased their trust in the federal government.

The dynamic interplay between social and political trust, as observed in both our descriptive and experimental analyses, suggests an intriguing pattern. The erosion of trust in institutions responsible for safeguarding citizens during a crisis is, in part, compensated by a concurrent increase in social trust. This behavior aligns with the “outward exposure” hypothesis, which is grounded in the more general “emancipation theory of trust” developed by Yamagishi et al.^27,28^ and extended to the context of a pandemic crisis by Gambetta and Morisi^8^. This hypothesis posits that, in times of crisis, individuals turn to interpersonal relationships as a coping mechanism when policy responses are perceived as inadequate or weak, leading to increased social trust.

## Background

There are competing hypotheses about trust dynamics in times of crisis. Regarding political trust, some studies suggest that, since governments are evaluated more strictly during extraordinary (especially negative) events^11,12^, citizens’ trust in institutions and politicians may vary depending on the perceived performance of these actors. Voters might, on the one hand, have lower political trust when authorities are deemed responsible for the crisis and/or for inadequate policy responses^29,30^. On the other hand, in the midst of a shared misfortune, voters might look for political stability, unity, and competence, and, therefore, their political trust might rise; in other words, when it comes to a fight against a collective threat, voters “rally round the flag”^13,31^. However, from a dynamic perspective, this rallying behavior may eventually give way to disillusionment when citizens’ perceptions on the effectiveness of governmental policies are revised downward due to personal experiences and additional information on the government’s performance. In essence, the initial rally effect can be followed by a decline in political trust when individuals become disenchanted with the crisis management and policy responses. Alternatively, it can be reinforced by positive perceptions of the political management of the crisis, thereby leading to higher political trust.

Regarding social trust, a strand of literature suggests that this specific type of trust is formed in childhood and changes only slowly thereafter due to experience^14,15^. Social trust tends, from this perspective, to be rather stable during shocks, whereas political trust is more volatile. In contrast, another strand of literature conceptualizes social trust as the belief that others behave trust-worthily^27,32,33^. By providing clues on the other’s behavior, contextual experiences can change individuals’ propensity to take the risk of dealing with strangers, and hence to trust others. Low generalized trust can emerge if, for instance, the pandemic makes self-regarding coping strategies more appealing. This can lead to social isolation, where people confine themselves within close-knit circles of acquaintances^34,35^. Low social trust could also emerge if the pandemic shock generates a perception of the failure of both institutions and society to effectively navigate the crisis^36^, yet positive information on social cohesion can restore it^37^. The pandemic shock may also jeopardize one’s own health, financial, and psychological resources: these are among the factors that are positively correlated with prosocial behavior^16,17,38,39^. Studies in post-disaster and post-conflict contexts highlight, however, an opposite pattern: social trust heightens, most likely because of increased empathy with unknown people^19,40^, increased social cohesion (people feel themselves to be “in the same boat”^18^), extended cooperation as a recovery strategy^20^, and an intensified search for social support outside one’s own network of trusted persons^8,27,28^. Based on the latter results, social trust can experience an upswing in the face of a deteriorating pandemic situation. Realizing that political institutions may not be functioning optimally could prompt citizens to seek alternative sources of support.

The empirical literature on the pandemic and trust mostly focuses on social and political trust separately and often offers contradictory findings (see Devine et al.^41^ for a review). Regarding trust in institutions, for instance, individuals experiencing COVID-19 in Spain showed lower levels of political trust^22^, whereas this type of trust seemed to be higher in European countries because of lockdown measures^42–45^, as well as in countries outside Europe^10^. However, most likely because of citizens’ changes in evaluations of health policies, this effect faded by the end of 2020^43^. Conversely, in the Netherlands, it has been argued that trust dynamics were influenced not so much by lockdown policies, but by a “rally ’round the flag” effect^9^. Similar results have been found in other countries^24,46,47^. In particular, the rally effect was mainly triggered by feelings of fear^46^ and perceptions of increased health risks^23,24,45^. Some of these findings also highlighted that satisfaction with governmental responses, together with the need for unity and political stability, emerged as the main factors that influenced political trust during the pandemic^48^. Other studies documented that the positive perception of the government’s response to the pandemic was key to sustain high levels of political trust^49–51^. In particular, Martinez-Bravo and Sanz^50^ showed that priming individuals with negative cues on regional pandemic management policies affected political trust.

With respect to social trust, Gambetta and Morisi^8^ showed that interpersonal trust increased in response to COVID-19 exposure in Italy, most likely because they became more dependent on other people’s support. Similar results were found in South Korea^49^. On the other hand, there is evidence that COVID-19 exposure decreased social trust in China^21,52^, and Malik et al.^53^ showed that people in the US were less trustful towards individuals wearing a face mask. Some studies investigate the effects of the COVID-19 pandemic on both social and political trust. Daniele et al.^25^ showed that in four European countries both social and political trust fell as a result of priming messages on COVID-19. However, their results are heterogeneous by the target of trust and the topic reported in their priming experiment. Liu et al.^26^ highlighted that, in China, both social and political trust decreased following greater exposure to COVID-19 risk at the regional level. Similarly, Brück et al.^54^ showed, using a sample from different countries, that being in contact with someone with COVID-19 symptoms decreased both social and political trust. Conversly, Esaiasson et al.^7^ found that, for Sweden, both interpersonal and institutional trust increased, even for groups that are politically distant from the ruling parties. This suggests that the rally effect might go beyond political affiliation.

The aforementioned studies on the COVID-19 pandemic predominantly focus on a rally effect observed at the onset of the crisis. Although this effect may result in initially elevated levels of social and political trust, our perspective introduces the possibility of a divergence over time. This viewpoint is supported by various studies demonstrating the waning of the rally effect beyond the earliest phases of the pandemic^9,24,48^, whereby the perception of the crisis (mis)management emerges as a more influential factor in determining trust^24,48^. Departing from previous research that acknowledges the correlation between institutional and social trust^55^, we propose a novel perspective on their interplay during an unforeseen shock. We predict a divergence between political and social trust driven by perceived institutional performance. Specifically, we hypothesize that individuals, feeling increasingly vulnerable to shocks and perceiving a lack of support from governmental institutions, will experience a decline in political trust. In the context of rising contagion rates and perceived crisis mismanagement, politically disillusioned citizens may seek support from the broader society, increasing their reliance on and trust in unknown individuals.

To test the hypothesis regarding the role of perceived crisis (mis)management in trust divergence, we conduct an information-provision experiment aimed at exogenously shifting individual perceptions of the effectiveness of anti-pandemic policies. We posit that exposure to negative (positive) messages on the country’s performance during the pandemic would prompt participants to decrease (increase) their trust in the government’s ability to safeguard citizens’ health and economic well-being. Reduced trust in the government might, in turn, motivate citizens to seek support beyond governmental channels. This heightened (lessened) outward search for support could consequently translate into a greater (lesser) need to trust unknown others as alternative sources of assistance.

Finally, we examine whether these dynamics vary by respondents’ political orientation in the US, and whether they mirror variations in attribution of responsibility about the pandemic crisis. A first hypothesis, based on the “hyper-accountability” theory^56^, suggests that Trump voters became more critical of the performance of the political forces they voted for, especially if they felt threatened by the pandemic. Disappointed Trump voters would reduce political trust more than non-Trump voters if they felt abandoned by the institutions from which they expected protection. If these voters—threatened by an unexpected crisis—look for support from other people, political disappointment can also translate into increased social trust, as observed in the aforementioned studies. For this reason, expanding upon the earlier hypotheses regarding citizens’ responses to positive or negative messages about the country’s performance, we posit that Trump voters, when exposed to a negative message, exhibit a stronger reaction compared to non-Trump voters. The former may experience heightened disappointment in how the crisis was managed by the political leader they have trusted, and from whom they anticipate protection against the health and economic challenges of the crisis. Consequently, their political (social) trust is revised downwards (upwards).

A second hypothesis, instead, would suggest an opposite pattern: when socio-economic uncertainty arises, identification with a political party may shape how individuals interpret reality. In periods of crisis, supporters of the ruling parties might evaluate policies in a way that is mostly consistent with their own political views^57,58^. Hence, the political trust of Trump voters might increase as they optimistically expect that the policies introduced by their leaders would benefit all citizens, and eventually protect their own interests^59^. Based on this hypothesis, in our experimental setting, we anticipate that Trump voters would dismiss the negative message on the country’s performance, deeming it inconsistent and incongruent with their pre-existing political beliefs. Conversely, when exposed to a positive message aligning with their priors, Trump voters are expected to exhibit a more pronounced reaction compared to non-Trump voters. This reaction would be driven by partisan-based motivated reasoning^57^, leading to heightened governmental judgments on how the crisis was managed by the political leader they trust. As a result, their political (social) trust undergoes an upward (downward) revision.

## Results

Before discussing the results, an immediate concern arises regarding their external validity due to the non-representative nature of the Amazon MTurk sample compared to the broader U.S. population. To address this issue, we conducted a reanalysis by incorporating post-stratification and attrition weights, thereby aligning our sample more closely with what would be considered representative of the U.S. population. We have documented the outcomes of this reanalysis in Section 7.1 of the Appendix, revealing only marginal adjustments in the estimated values.

### Social and political trust dynamics and exposure to COVID-19

Table R1 in the Appendix features a fixed-effects panel model, in which we regress measures of institutional trust on wave indicators, weighting for attrition. Here, we focus mainly on trust in the federal government to ensure compatibility with experimental results. Results on trust towards other institutional targets are in Appendix, where we consider all institutional targets both separately and together, i.e., aggregated through the first component of a principal component analysis.

**Figure 1:**
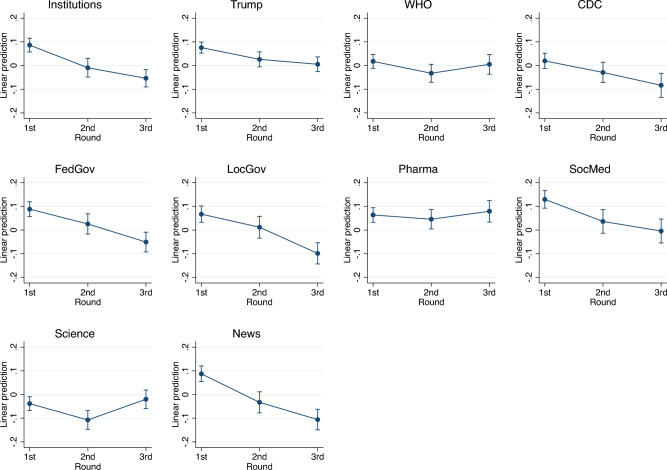
Institutional trust dynamics.

Variables are standardized, and coefficients represent the change in standard deviations of trust from the first wave. Figure 1 shows regression predictions, while Figure A9 in Appendix shows a graphical representation of coefficients. We observe that trust in institutions declines over time. An exception is trust in science which, in the third wave, went back to the values of the first wave. Results for attribution of responsibility are discussed in Appendix (Section 3).

**Figure 2:**
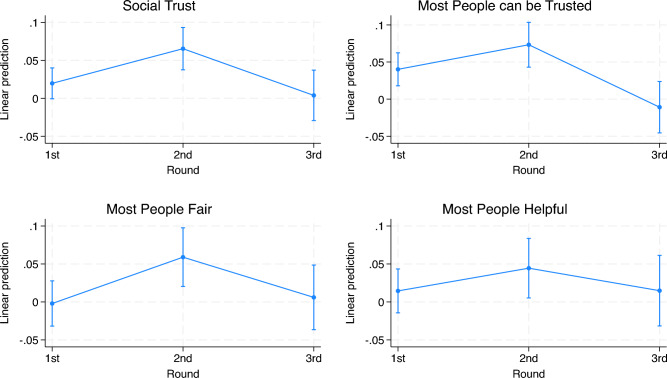
Social trust dynamics.

We also estimate the trend in social trust with the same fixed-effects panel model. Table R3 in Appendix, jointly with Fig. 2 (and Figure A11), shows that social trust was quite steady, with a slight increase in wave two. In Table 1, we repeat the previous analysis, also controlling for indirect and direct COVID-19 exposure. Including controls for COVID-19 exposure does not change coefficients on wave indicators significantly. We consider two different measures of COVID-19 exposure: (1) the number of deaths per-capita, at the time of the response, in the panelist’s county (this measure accounts for indirect COVID-19 exposure); (2) whether the respondent, one of their relatives or friends got infected with COVID-19 at the time of the response (this measure accounts for a direct encounter with COVID-19). While indirect exposure plays a negative role in social trust, direct exposure positively correlates with it. This suggests that the first measure may capture positive personal experiences with doctors or help and kindness received from others^60,61^, whereas the second relates more to perceived health risks or worries about the pandemic^61^.

This descriptive analysis uncovers a potential “rally” behavior that occurred either before or during the first survey wave, notably before the pandemic peaked. During this period, political trust was high, resulting in a relatively low outward search for support, as indicated by the low levels of social trust. However, these trends reversed as the pandemic peaked, indicating a downward revision of citizens’ evaluations of governmental performance. This revision triggered an increased need for support from others, manifested in the rising levels of social trust.

**Table 1:** Social and political trust dynamics.

	(1)	(2)	(3)	(4)
	Trust in the Federal Government		Social trust	
Wave 2	$-$0.0794**	$-$0.0604*	0.0621***	0.0426*
	(0.0375)	(0.0358)	(0.0238)	(0.0225)
Wave 3	$-$0.205***	$-$0.120***	0.0393	$-$0.0415*
	(0.0479)	(0.0348)	(0.0342)	(0.0251)
Cumulative COVID-19 Deaths	0.0537*		$-$0.0422**	
	(0.0279)		(0.0199)	
Direct COVID-19 Exposure		$-$0.106		0.143***
		(0.0739)		(0.0476)
Constant	0.108***	0.104***	0.00311	$-$0.00109
	(0.0205)	(0.0191)	(0.0137)	(0.0128)
Observations	2,272	2,283	2,272	2,283
R-squared	0.015	0.015	0.009	0.015
Number of wid	966	973	966	973
Test Wave 3 - Wave 2=0	$-$0.126***	$-$0.0596	$-$0.0228	$-$0.0841***
	(0.0426)	(0.0369)	(0.0305)	(0.0272)

Columns (1)–(2) use trust in the Federal Government as a dependent variable, while columns (3)–(4) use a pca variable of social trust, comprising three items for the evaluation of generalized social trust. Dependent variables are standardized; coefficients represent outcome’s change in terms of standard deviations. Robust standard errors in parentheses; ***p<0.01, **p<0.05, *p<0.1.

### Social and political trust dynamics and partisanship

**Figure 3:**
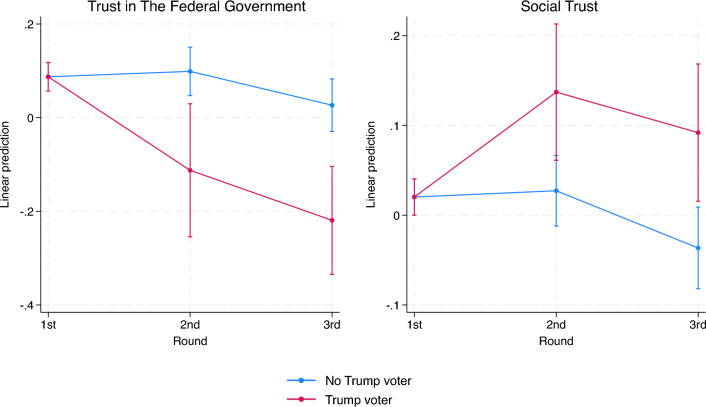
Trust dynamics by voting behavior.

**Figure 4:**
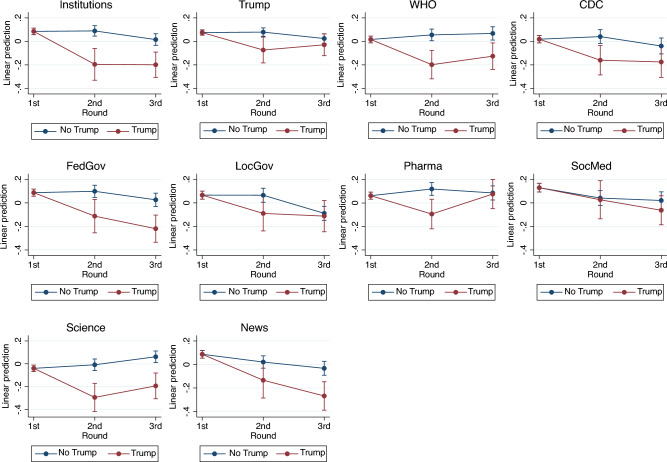
Partisan dynamics of institutional trust.

We use a fixed-effects panel regression to model the dynamics of our political and social trust measures, allowing for differential trends by political affiliation. Figure 3 displays a clear partisan trend (Figure A12 and Table R6 in Appendix): Trump voters’ trust in the Federal Government fell (especially in the second wave), while their social trust, which remained stable in wave 3, grew. Non-Trump voters showed a slight reduction in social and political trust only in the third wave. These results suggest that the dynamics observed in the previous section, i.e., the reduction in trust in the Federal Government and the (mild) increase in social trust, were mainly driven by Trump voters. Finally, Fig. 4 shows that the decline in political trust for Trump voters extends to all the institutional targets considered in the survey, except for social media (Table R8 and Figure A13 in Appendix). Importantly, among governmental institutions, Trump voters lost trust in Trump and the Federal Government but not local governments. This pattern should be considered jointly with results from Table R9 in Appendix: Trump voters were more likely to attribute responsibility to Trump in the second survey wave (during the peak of the first pandemic wave). This mirrored the fall in their political trust. These voters, on average, blamed the Federal Government less and Chinese actors more, which is consistent with their nationalistic preferences and, potentially, differential media exposure. These results again suggest that they might have felt unprotected by institutions, and hence looked for support elsewhere in society. Trump voters were, in fact, among those respondents who were least likely to attribute responsibility to the US public for the pandemic (Table R9 and Figure A7 and A14 in Appendix).

To assess whether this behavior is related to a heightened perception of pandemic risks, we test whether Trump voters exposed to COVID-19 reacted differently from non-Trump voters. Results in Table 2 show that Trump voters directly exposed to COVID-19 were more likely to see their trust in the Federal Government fall and their social trust rise than non-Trump voters exposed to COVID-19. Expanding our analysis to encompass a broader measure of trust in institutions reveals an even stronger negative correlation between direct COVID-19 exposure and institutional trust among Trump voters (Table R5 in Appendix). A similar pattern emerges when considering indirect COVID-19 exposure, with a decline in political trust and an increase in social trust for Trump voters, though the latter effect did not reach statistical significance. These results suggest that the worries about the pandemic, proxied by the number of COVID-19 deaths in a given county, contribute to political dissatisfaction, particularly among Trump supporters. However, it is mainly when these supporters have been directly exposed to the virus, and hence potentially experience kindness and help from others^37^, that their political dissatisfaction translates into an outward search for support, resulting in an increase in social trust.

The substitution dynamic of political trust with social trust resulting from political dissatisfaction with the pandemic management holds just at the aggregate level, while the link between the two types of trust weakens for Trump voters in within-individual regressions. More specifically, in additional estimates, we use Social Trust as the dependent variable controlling for Trust in the Federal Government interacted with the Trump-voter indicator. Results (Section 6, Appendix) indicate that, for non-Trump voters, the two trends in trust are positively correlated ($\beta =0.0981; p=0.008$), aligning with established findings in the literature on the positive correlation between social and political trust^55^. However, the correlation is close to zero for Trump voters. This finding provides additional support for the hypothesized mechanism driving the divergence in social and political trust during a crisis at the aggregate level. Dissatisfaction with the institutions and political forces expected to provide support leads to a decline in political trust and, hence, an increase in social trust. In essence, disillusioned citizens, particularly Trump voters in our context, play a pivotal role in steering this nuanced shift by actively seeking alternative sources of support within society, thereby attenuating the positive correlation between political and social trust observed at the societal level.

Admittedly, an alternative interpretation could be that Trump voters became less prone to adhere to social distancing (or implement other forms of protective behavior) and were more open to social interactions. These features may explain the increase in social trust and the decrease in political trust found in previous estimates. This kind of behavior, however, might also have increased direct exposure to COVID-19. While the experimental results partially rule out this alternative mechanism, additional results reported in Section 4 of the Appendix dismiss the possibility that COVID-19 exposure is driven by the aspects of political affiliation correlated with social and political trust.

### The survey experiment

In the third round, we conducted a randomized survey experiment based on information provision to allow a causal interpretation of the panel results. This experimental design also enables a deeper analysis of a specific mechanism that underlies the observed trust dynamics—namely, the influence of information about the country’s performance in managing the pandemic crisis on individuals’ perceptions. Respondents were randomly assigned to three groups. The first group received a positive message on the pandemic crisis, explaining the economic and health improvements achieved by the US in November, relative to April 2020. A second group received a negative message, reporting how the US underperformed economically with respect to 2019, and witnessed the worst health consequences compared with the rest of the world. Finally, a third group received no message (control group). The exact wording of these messages is reported in Table 3. In addition to the trust measures described earlier, respondents were asked questions about their own opinions regarding the economic and public health situation, as well as on the perceived valence of the stimulus, which we used to ensure that our manipulations were effective.

**Table 2:** Social and political trust by exposure to COVID-19 and political orientation.

	(1)	(2)	(3)	(4)
	Trust in the Federal Government		Social trust	
Wave 2	$-$0.0785**	$-$0.0613*	0.0619***	0.0431*
	(0.0374)	(0.0357)	(0.0238)	(0.0225)
Wave 3	$-$0.201***	$-$0.126***	0.0387	$-$0.0374
	(0.0481)	(0.0341)	(0.0343)	(0.0252)
Direct COVID-19 Exposure		0.00102		0.0761
		(0.0614)		(0.0562)
Direct COVID-19 Exposure*Trump voter		$-$0.250*		0.157*
		(0.149)		(0.0926)
Cumulative COVID-19 Deaths	0.0797***		$-$0.0469**	
	(0.0245)		(0.0221)	
Cumulative COVID-19 Deaths*Trump voter	$-$0.0988**		0.0177	
	(0.0503)		(0.0289)	
Constant	0.103***	0.111***	0.00406	$-$0.00602
	(0.0208)	(0.0210)	(0.0137)	(0.0134)
Observations	2,272	2,283	2,272	2,283
R-squared	0.019	0.019	0.009	0.018
Number of wid	966	973	966	973
Test Wave 3–Wave 2=0	$-$0.123***	$-$0.0652*	$-$0.0233	$-$0.0805***
	(0.0430)	(0.0360)	(0.0305)	(0.0272)

Columns (1)–(2) use trust in the Federal Government as a dependent variable, while columns (3)–(4) use a pca variable of social trust, comprising three items for the evaluation of generalized social trust. Dependent variables are standardized; coefficients represent outcome’s change in terms of standard deviations.Robust standard errors in parentheses; ***p<0.01, **p<0.05, *p<0.1.

Results show that respondents willing to vote for Trump in the 2020 US presidential election displayed, on average, higher trust in the Federal Government (Table 4). However, the coefficient of the interaction between the Trump-voter and the negative message indicators is negative and significant. When exposed to negative information about the pandemic, Trump voters displayed increased social trust and decreased political trust. This supports the concept of an “institutions-people substitution” dynamic, as observed in the longitudinal results, reinforcing our interpretation of the aforementioned panel findings: when citizens perceive the government as untrustworthy and incompetent in managing a national crisis, their political trust decreases, leading to a higher need for informal support and, consequently, increased reliance on trusting other individuals. This piece of evidence also supports the aforementioned “hyper-accountability” hypothesis.

**Table 3:** Experimental treatments: positive and negative messages.

Negative	Positive
Till October 20, 2020, the United States had 8.26 million confirmed cases of COVID-19 and over 220,000 COVID-19 deaths (U.S. CDC, 2020). While the US has 4% of the world population, it accounts for around 20% of confirmed COVID-19 cases and deaths in the world. During the COVID-19 pandemic, the United States recorded its largest quarterly drop in economic output on record, a decrease of 9.1% in the second quarter of 2020 (compared to the first quarter). To put this contraction into a historical context, the second steepest drop in quarterly GDP since 1947 was during the 2007-2009 recession of 3%. Moreover, in April 2020, the monthly unemployment rate was 14.7%, compared to 3.5% in February 2020 (U.S. Census Bureau 2020).	In the United States, the current case fatality rate for COVID-19 has dropped to around 2.7% in October compared to the peak of 6.1% in May. (Case fatality rate is the percentage of death cases diagnosed of COVID-19 over total confirmed cases of COVID-19). Only around 5% of the viral tests are positive in October, while around 20% of the viral tests were positive in late April (U.S. CDC 2020). Ten million workers have found jobs since the high point of unemployment in April. Moreover, in April 2020 the U.S. personal savings rate reached its highest recorded level. Retail sale also has been growing since early May: by August, retail sales were 2.6 percent above their August 2019 level (U.S. Census Bureau 2020).

The control group received no message.

**Table 4:** Social and political trust by political orientation: experimental results.

	(1)	(2)	(3)	(4)
	Baseline		Interaction with Trump voter dummy	
	Social Trust	Trust in the Federal Government	Social Trust	Trust in the Federal Government
Positive Priming	$-$0.0303	0.00622	$-$0.0454	0.0527
	(0.0363)	(0.0571)	(0.0448)	(0.0658)
Negative Priming	$-$0.0397	$-$0.0889	$-$0.101**	$-$0.00802
	(0.0383)	(0.0593)	(0.0506)	(0.0716)
Trump voter			0.0633	0.328***
			(0.0562)	(0.103)
Positive Priming*Trump voter			$-$0.00572	$-$0.195
			(0.0832)	(0.133)
Negative Priming*Trump voter			0.143*	$-$0.277**
			(0.0812)	(0.138)
Cumulative COVID-19 Deaths	$-$0.0152	0.0207	$-$0.0176	0.0265
	(0.0153)	(0.0276)	(0.0156)	(0.0282)
Direct COVID-19 Exposure	0.00616	$-$0.0113	0.00862	$-$0.0165
	(0.0323)	(0.0546)	(0.0332)	(0.0558)
Took part in the previous waves	$-$0.0524	$-$0.697	$-$0.115	$-$1.254***
	(0.120)	(0.455)	(0.121)	(0.178)
Social Trust (pre-experiment)	0.881***		0.874***	
	(0.0148)		(0.0160)	
Political Trust (pre-experiment)		0.632***		0.596***
		(0.0271)		(0.0313)
Constant	0.143	0.180	0.283	0.940*
	(0.261)	(0.645)	(0.278)	(0.487)
Observations	964	965	909	910
R-squared	0.789	0.473	0.786	0.477
Test Negative Priming–Positive Priming=0	$-$0.00944	$-$0.0951	$-$0.0556	$-$0.0607
	(0.0364)	(0.0581)	(0.0441)	(0.0718)
Test (Negative Priming–Positive Priming)*Trump=0			0.149*	$-$0.0824
			(0.0838)	(0.132)

Robust standard errors in parentheses; ***p<0.01, **p<0.05, *p<0.1. The dependent variable in column (1) and (3) represents the extent to which respondents believe that “most people can be trusted” versus “you cannot be too careful”. The dependent variable in columns (2) and (4) represents how much respondents think that the Federal Government can be trusted to do what is right. Dependent variables are standardized; coefficients represent outcome’s change in terms of standard deviations. Regressions control for socio-demographic characteristics including ethnicity, employment status, education and income. Additional controls include indirect (the number of deaths per-capita, at the time of the response, in the panelist’s county) and direct (whether the respondent or one of their loved ones got a COVID-19 diagnosis) COVID-19 exposure, a panel dummy indicating if the respondent was part of the panel. Finally, a pre-experimental measure of the dependent value is added. For social trust, it is a pca variable including the three items asked in the panel. Political trust is the result of pca on trust in Trump, the Federal Government, and Local Governments.

The study found no significant effects of the positive message. One plausible explanation is that, by the third round, citizens may have become accustomed to positive pandemic narratives, diminishing their responsiveness. Furthermore, individuals’ responses to information provision might be biased by partisan-motivated reasoning, depending on whether the tone of the message aligns with political orientations. However, in that case, we should observe a larger (no/small) impact of the positive (negative) message for Trump voters if motivated reasoning made them perceive the negative (positive) message as less negative (more positive); this should generate a change in their political trust only/mainly in response to an ideologically congruent piece of information, i.e., the positive message. Our results document an opposite pattern. Despite partisan-motivated reasoning affecting perceptions of the message’s tone (see Sections 5 and 7.3 in Appendix), it doesn’t consistently influence trust responses. Notably, Trump voters revised their political trust downward after the negative pandemic message, suggesting that motivated reasoning might not be the driving mechanism in this context. If anything, it should downward bias the estimated effects of the negative message.

## Robustness checks

Rather than capturing the political leaning of respondents, the difference between Trump and non-Trump voters might entail differences in socio-demographic characteristics between the two groups. To mitigate this concern, we replicate our main estimates (Table 2), replacing the ‘Trump Voter’ dummy with the residuals from a logistic regression, where the probability of being a Trump voter is used as the dependent variable. Here, the results confirm that political trust decreased while social trust increased for individuals with higher residuals; however, we do not find changes for other voters (see Section 7.2 in Appendix for further details).

Another potential concern is the validity of the stimuli used in the experiment. We verified whether the messages shown to participants could tile this effect through manipulation checks, and found that this was the case (see Section 7.3 in Appendix). An additional manipulation check provides further evidence that participants in the positive-message group perceived the positive message as conveying a positive sentiment, while those in the negative-message group accurately discerned the pessimistic tone of the text they read. Moreover, the positive message wielded a more pronounced influence than the negative one, with the latter being perceived as comparatively less negative than the positivity perceived in the positive message (Section 7.3 in Appendix). Such perception asymmetry, however, is not a concern for our estimates: it should only induce a downward bias in the negative-message effect.

## Conclusion

This paper investigates how trust evolved during the first pandemic wave in the United States. Contrary to political trust, social trust increased, following patterns observed in European studies^8^. Importantly, in the context of a highly polarized political landscape like that of the US, this dynamic was mainly driven by disappointed Republicans, especially when directly exposed to COVID-19. These results align with the literature arguing that trust in others may increase during crises, as vulnerable citizens or those in need seek support beyond their immediate networks^27,28^. Concurrently, our results support the hypothesis that political trust decreases when institutional authorities are perceived as either responsible for the crisis or inadequate in their response^29,30^.

The experimental part of the survey provides causal evidence of a potential mechanism behind these patterns: the perceived institutional performance in managing the pandemic. Relative to Democrats, when exposed to a negative narrative on the country’s performance during the crisis, Republicans reported lower trust in the Federal Government and higher trust in other people. This confirms the inverse relationship between social and political trust highlighted in the descriptive panel estimates, where these two types of trust diverged when the pandemic peaked. When Republicans, who express disappointment with their political leader’s handling of the crisis, are exposed to negative narratives about the crisis, they tend to shift their reliance from political institutions to individuals for support.

In conclusion, our results show that dissatisfaction with political responses to a common threat can lead people to seek support beyond their immediate networks and institutions, leading to increased trust in others. Hence, when perceived risks arise, social trust might act as a compensatory mechanism for the lack of political trust, with politically disappointed citizens potentially substituting institutions with interpersonal trust when seeking support.

## Methods

This project was approved by the Bocconi Research Ethics Committee (No. FA000075). All experiments were performed under relevant named guidelines and regulations. Informed consent was obtained from all participants.

The longitudinal study follows a panel of 974 Amazon MTurk respondents from the US, through three waves (Section 1 in Appendix). The sample starts with a panel of 1041 participants; 67 participants (6.44%) were dropped because they failed one of the five attention checks inserted at different points throughout the three waves. The first round was conducted from April 6th to April 16th 2020; the second wave was collected between April 29th and May 22nd 2020; the last round occurred from October 21st to November 2nd 2020, just before the US presidential elections.

Respondents were asked about their sociodemographic characteristics during the first wave, including age, gender, race, household composition, education, employment, income, health, political ideology and their voting affiliation in the 2016 elections. The survey included questions on trust, jointly with experiences of, and opinions about the COVID-19 pandemic, and repeated in all three survey waves. The trust section of the survey measured, on a scale from one (1) to five (5), trust towards other people through questions widely used in other surveys (Section 1 in Appendix). Trust towards institutions was asked on a scale from one (1) to five (5), and included: the (former) President of the United States (Donald Trump); social media; Centers for Disease Control and Prevention (CDCs); the mass media; scientists; WHO; pharmaceutical companies; and local and federal government. In the COVID-19 section, respondents were asked whether they, their family, friends, or acquaintances have been diagnosed with COVID-19 and questions on the attribution of responsibility for COVID-19. The order of COVID-19 and trust block was randomized. Sample statistics are reported and discussed in Section 1 of the Appendix (Table A2 and A3), where we also report a detailed description of all variables (Table A3).

The first outcome variable we consider is Trust in the Federal Government. In Appendix, we also show results for a synthetic measure of *institutional trust*, obtained by extracting the first factor from a principal component analysis, including trust towards all institutional targets we elicited in the survey. The other key outcome variable is *social trust*, obtained through a principal component analysis of the three questions measuring trust in other people.

A total of 504 respondents were present in all waves; 17.04% of respondents left the sample after the first wave, while 22.07% dropped out in the third wave. A share of 9.14% of respondents was absent in the second wave but returned in the third. For this reason, all estimates control for attrition through the inverse-probability weighting strategy (Section 2 in Appendix).

### Supplementary Information

Supplementary Information.

## Data Availability

Data and programs are available at the following website: https://osf.io/mxgzr/?view_only=a08a8ad849db4728a3c9c68ccd24c75d.
