# Supplementary material for: Social and political trust diverge during a crisis
Source: Sci Rep. 2024 Jan 3;14:331. doi: 10.1038/s41598-023-50898-4 (PMC10764309; doi:10.1038/s41598-023-50898-4)
Supplement: Supplementary file 1 — Supplementary Information. [file 41598_2023_50898_MOESM1_ESM.pdf]

ONLINE APPENDIX

Table of contents

1. DATA AND DESCRIPTIVE STATISTICS 3
2. ATTRITION MODEL 3
3. RESULTS ON ATTRIBUTION OF RESPONSIBILITY 4
4. PARTISAN TRENDS IN COVID-19 EXPOSURE 4
5. HETEROGENEITY BY SOCIAL-MEDIA EXPOSURE 4
6. CORRELATION BETWEEN SOCIAL AND POLITICAL TRUST 5
TABLE S1. WITHIN-SAMPLE CORRELATION BETWEEN POLITICAL TRUST AND SOCIAL TRUST (ATTRITION WEIGHTS) 5
7. ADDITIONAL ROBUSTNESS CHECKS 6
7.1 POST-STRATIFICATION WEIGHTS 6
7.2 USING AN ALTERNATIVE MEASURE FOR BEING A TRUMP VOTERS 6
7.3 THE EFFECTS OF OUR STIMULI ON OPINIONS AND PERCEPTIONS 7
ADDITIONAL REFERENCES 8
MAIN REGRESSION TABLES 9
TABLE R1. INSTITUTIONAL TRUST DYNAMICS 9
TABLE R2. DYNAMICS OF ATTRIBUTION OF RESPONSIBILITY 9
TABLE R3. DYNAMICS OF SOCIAL TRUST 10
TABLE R4. DYNAMICS OF SOCIAL TRUST AND INSTITUTIONAL TRUST 10
TABLE R5. DYNAMICS OF SOCIAL TRUST AND INSTITUTIONAL TRUST BY EXPOSURE TO COVID AND POLITICAL ORIENTATION 11
TABLE R6. PARTISAN TRUST DYNAMICS 11
TABLE R7. DYNAMICS OF SOCIAL TRUST AND INSTITUTIONAL TRUST BY PARTISANSHIP 12
TABLE R8. PARTISAN DYNAMICS OF INSTITUTIONAL TRUST 12
TABLE R9. PARTISAN DYNAMICS OF ATTRIBUTION OF RESPONSIBILITIES 13
ADDITIONAL TABLES 14
TABLE A1. DESCRIPTION OF VARIABLES 14
TABLE A2. DESCRIPTIVE STATISTICS, SOCIO-DEMOGRAPHIC CHARACTERISTICS. 15
TABLE A3. DESCRIPTIVE STATISTICS, CONTINUOUS VARIABLES 15
TABLE A4. ATTRITION 15
TABLE A5. ATTRITION LOGIT MODEL 16
TABLE A6. DYNAMICS OF BEING EXPOSED TO COVID-19. 17
TABLE A7. MODEL TABLE R1 WITH POST-STRATIFICATION WEIGHTS 18
TABLE A8. MODEL TABLE R2 WITH POST-STRATIFICATION WEIGHTS 18
TABLE A9. MODEL TABLE R3 WITH POST-STRATIFICATION WEIGHTS 19
TABLE A10 MODEL TABLE 2 WITH POST-STRATIFICATION WEIGHTS 19
TABLE A11 MODEL TABLE R6 WITH POST-STRATIFICATION WEIGHTS 20
TABLE A12 MODEL TABLE R8 WITH POST-STRATIFICATION WEIGHTS 20
TABLE A13 MODEL TABLE R9 WITH POST-STRATIFICATION WEIGHTS 21
TABLE A14 MODEL TABLE 3 WITH POST-STRATIFICATION WEIGHTS 21
TABLE A15 MODEL TABLE 4 WITH POST-STRATIFICATION WEIGHTS 22
TABLE A16 MODEL TABLE A17 WITH POST-STRATIFICATION WEIGHTS 23

TABLE A17 MODEL TABLE 4 (COLUMN (3)-(4)) SPLIT BY SOCIAL MEDIA EXPOSURE 24

ADDITIONAL FIGURES 25

FIGURE A1 MODEL FIGURE 1 WITH POST-STRATIFICATION WEIGHTS 25
FIGURE A2 MODEL TABLE R2 25
FIGURE A3 MODEL FIGURE A2 WITH POST-STRATIFICATION WEIGHTS.. 26
FIGURE A4 MODEL FIGURE 2 WITH POST-STRATIFICATION WEIGHTS 26
FIGURE A5 MODEL FIGURE 3 WITH POST-STRATIFICATION WEIGHTS (PANEL A) AND ALTERNATIVE VERSION WITH REPRESENTATION OF COEFFICIENTS (PANEL B).. 27
FIGURE A6 MODEL FIGURE 4 WITH POST-STRATIFICATION WEIGHTS 28
FIGURE A7 MODEL TABLE R9 28
FIGURE A8 MODEL FIGURE A7 WITH POST-STRATIFICATION WEIGHTS.. 29
FIGURE A9. GRAPHICAL VISUALIZATION OF COEFFICIENTS FROM TABLE R1 29
FIGURE A10. GRAPHICAL VISUALIZATION OF COEFFICIENTS FROM TABLE R2 30
FIGURE A11. GRAPHICAL VISUALIZATION OF COEFFICIENTS FROM TABLE R3 30
FIGURE A12. GRAPHICAL VISUALIZATION OF COEFFICIENTS FROM TABLE R6 31
FIGURE A13. GRAPHICAL VISUALIZATION OF COEFFICIENTS FROM TABLE R8 31
FIGURE A14. GRAPHICAL VISUALIZATION OF COEFFICIENTS FROM TABLE R9 32
FIGURE A15. DYNAMICS OF TRUST IN THE FEDERAL GOVERNMENT BY UNEXPLAINED PROBABILITY OF BEING TRUMP VOTER 32
FIGURE A16. DYNAMICS OF SOCIAL TRUST BY UNEXPLAINED PROBABILITY OF BEING TRUMP VOTER 33
FIGURE A17. EFFECT OF DIRECT COVID-19 EXPOSURE ON TRUST IN THE FEDERAL GOVERNMENT BY UNEXPLAINED PROBABILITY OF BEING A TRUMP VOTER 33
FIGURE A18. EFFECT OF DIRECT COVID-19 EXPOSURE ON SOCIAL TRUST BY UNEXPLAINED PROBABILITY OF BEING A TRUMP VOTER 34
FIGURE A19. IMPACT OF PRIMING CONDITIONS ON THE PERCEPTION OF THE HEALTH AND ECONOMIC SITUATION 35
FIGURE A20. POSITIVE VS NEGATIVE MESSAGE PERCEPTION OF PRIMING.. 35
FIGURE A21. PARTISAN EFFECTS OF PRIMING CONDITIONS ON THE PERCEPTION OF THE HEALTH AND ECONOMIC SITUATION 36
FIGURE A22. POSITIVE VS NEGATIVE MESSAGE PERCEPTION OF PRIMING, BY POLITICAL ORIENTATION 37

1. Data and descriptive statistics

The survey data are collected through Amazon Mechanical Turk (MTurk), i.e. a platform that allows requesters to distribute tasks to a workforce who could perform them virtually. It is commonly used to distribute surveys for academic research because it allows for the recruitment of samples in short time, that, even if they are not nationally representative, have demonstrated solid reliability[1]. In particular, 93.56 percent of our panel sample passed the attention checks. This is in line with previous research which shows that MTurk workers are highly attentive responding to surveys[2]. Supplementary materials, including questionnaires and the survey flow are available at the following repository: https://osf.io/mxgzr/ .

Respondents were asked about their sociodemographic characteristics during the first wave, including age, gender, race, household composition, education, employment, income, health, political ideology and their voting affiliation in the 2016 elections. Some additional individual characteristics were collected during the second and third wave. Baseline sociodemographic information was collected only once during the study. In the second wave new questions on political ideology and wealth were asked, while the third round featured additional questions on income, media use, voting intentions and opinions on income inequality (not used in this study). The survey included questions on trust, jointly with experiences of, and opinions about the COVID-19 pandemic and repeated in all the three waves of the survey. The trust section of the survey measured, on a scale from one (1) to five (5), social trust through the following standard questions: "Generally speaking, would you say that most people can be trusted, or that you can't be too careful in dealing with people?", "Do you think that most people would try to take advantage of you if they got the chance, or would they try to be fair?", and "Would you say that most of the time people try to be helpful or that they are mostly looking out for themselves?". These questions have been widely used in other surveys, e.g., the European Social Survey[3]. Trust towards institutions was asked on a scale from one (1) to five (5), and included: the President of the United States (Donald Trump, in all waves); social media; Centers for Disease Control and Prevention (CDCs); the mass media; scientists; WHO; pharmaceutical companies; and local and federal government. In the COVID-19 section, respondents were asked whether they, their family, friends or acquaintances have been diagnosed with COVID-19 and whether they work in the health sector, how much they comply with specific forms of health-protective behavior, and questions on the attribution of responsibility for COVID-19. The order of questions was randomized across respondents. Some of them received first the COVID section before the trust section, while others received surveys following the opposite order. This ordering is assigned in wave one and it is maintained right through the survey.

Sample socio-demographic characteristics are reported in Table A2. The age of respondents ranges from 19 to 75, with an average of 38.33. There are more male than female respondents (56.98% vs 42.71%); most respondents are white, and the best represented ethnic minorities are Hispanic, Black, and Asian; almost half of respondents have a bachelor's degree while only 14.58% of respondents have a higher level of education. As to political leaning, the biggest group of respondents voted for Hillary Clinton in the 2016 elections (41.07% of the sample) while 22.9% voted "Other" candidates in the 2016 election, which underrepresents Trump voters as is often the case with MTurk surveys. Lastly, 13.38% of respondents reported having had a *direct* exposure to COVID-19, i.e. either the respondent, a member of their family or a friend have been diagnosed with COVID-19. Table A3 contains descriptive statistics of continuous variables, i.e. cumulative COVID-19 related deaths in the respondent's county, institutional trust, social trust and attribution of responsibility.

2. Attrition model

Table A4 shows that 504 respondents were present in all waves, 17.04% of respondents left the sample after the first wave, while 22.07% of respondents left the sample in the third wave. There is a share of 9.14% of

respondents who did not complete the survey in the second wave, but who were back for the third. For this reason, in all estimates, we control for attrition through the inverse-probability weighting strategy.
More specifically, since attrition could be a possible source of bias, in all models, we weighted the sample for attrition using inverse probability weighting. We weighted each observation by the inverse probability that the respondent participates in all survey waves. Hence, higher weights are assigned to respondents that are more likely to drop out of the survey at some point across waves. We used an attrition logit model to estimate the probability of non-attrition, i.e. the probability that a respondent takes part in all waves of the survey. The attrition model uses respondents' baseline characteristics to explain attrition. Age, gender, ethnicity, religion, education, health status, income, voting in 2016, social and institutional trust in the first non-missing wave are used as explanatory variables; the R^2 of the model is 0.2564. Results of the model are displayed in Table A5.

3. Results on attribution of responsibility

Table R2, Figure A2 and Figure A10 show results for the same fixed-effects panel model applied to variables measuring the extent respondents attribute responsibility to a specific actor, reported in column title. We can observe a general decline in the attribution of responsibility to all actors in wave two. Attribution of responsibility to the Chinese Government and the Chinese public reduced in the third wave, and the same occurred for attribution of responsibility to Trump. In the third wave, respondents started to attribute more responsibility to the US Government, WHO and the US public, while responsibility of CDCs, after a decline in wave two, went back up to the first wave's values. In general, respondents started to shift blaming towards domestic actors at the onset of the second pandemic wave.

4. Partisan trends in COVID-19 exposure

An alternative interpretation of results in Table 3 in the paper hinges on the possibility that Trump voters might have become less prone to adhere to social distancing (or to implement other forms of protective behavior) and were more open to social interactions. These features may explain the increase in social trust and the decrease in institutional trust found in previous estimates. This kind of behavior, however, might also have increased direct exposure to COVID-19. While the experimental results partially rule out this alternative mechanism, results in Table A6 help in excluding that COVID-19 exposure is driven by the aspects of political affiliation that are correlated with social and political trust.
Through a panel fixed-effects linear probability model, we predict the likelihood that a respondent received a COVID-19 diagnosis, and that either the respondent herself, one of her relatives or friends had been diagnosed with COVID-19. We observe that a better predictor of direct COVID-19 exposure is wave three, most likely because the more time having passed since the beginning of the pandemic, the most likely it is to report direct exposure to (or contact with persons who contracted) the virus. The probability that the respondent had been diagnosed with COVID-19 is also slightly influenced by the strictness of containment measures (strictness of containment policies is measured by the Oxford COVID-19 Government Response Tracker Containment and Health Index, which considers the presence and extent of restrictions, closures, testing, contact tracing, healthcare investments and investments in a vaccine.). Trump voters, however, do not show a different pattern of infection than non-Trump voters. This suggests that, in our sample, there are no partisan-specific trends in COVID-19 exposure.

5. Heterogeneity by social-media exposure

Social media were used intensively as a source of information during the pandemic[4,5]; importantly, they also played a significant role in shaping socio-economic and political attitudes[6-11]. On the one hand, heavy

social-media users may be more sensitive to information provision than less intensive users. First, their social-media platforms are frequently fed with news articles/comments on salient topics, and therefore they might be more receptive (and responsive) to information provision. Second, they might change or reinforce their own ideas depending on, among other factors, whether (and how much) the information received matches with their own prior beliefs, the level of endorsement it received and how polarizing the topic is[12]. On the other hand, we might also conjecture that intensive social-media users are less sensitive to information provision on a very popular topic. They might have been exposed to a large number of pandemic-related articles and opinions prior to our experiment. As such our messages might not be powerful enough to alter their consolidated beliefs or to induce significant emotional reactions.
We re-estimate the model in Table 4 in the paper separately for heavy and not-heavy social media users. The former group includes participants with an above-median score for frequent social-media use, considering the overall time they usually spend on Twitter, Facebook, Instagram, YouTube, Reddit and Tiktok (respondents were asked the frequency they used each of a series of social media throughout the week on a scale from 1 to 8; we calculated for frequency of social media use by summing the frequency of all considered social media). Results show that our main findings are driven by intensive social-media users (Table A17). The decrease (increase) in political (social) trust in response to negative information on the pandemic was mainly driven by the share of Trump voters who were exposed particularly to social media (in our sample.
These results suggest that the two hypothesized effects for intensive social-media users may coexist, i.e. both habituation and responsivity to social-media content might be at play. Habituation might occur most likely for news articles that are congenial to one's own political ideology (i.e. the positive message for Trump voters in our experiment) as social-media users might consume more news articles that are aligned their political views, whereas responsivity emerges mainly for news articles that are not aligned with one's own prior beliefs (i.e. the negative message for Trump voters in our setting).
In the Section 7.3 below, we provide suggestive evidence that the changes in trust induced by the uncongenial message for Trump voters are likely driven by an emotional response rather than by a change in beliefs.

6. Correlation between social and political trust

The substitution dynamic of political trust with social trust resulting from political dissatisfaction with the pandemic management is further confirmed in additional estimates where the dependent variable is Social Trust and where we control for Trust in the Federal Government, interacted with the Trump voter indicator. Results are reported in the Table below and indicate that, on average, the two trends in trust are positively correlated for non-Trump voters, aligning with established findings in the literature on the positive correlation between social and political trust. However, the correlation disappears for Trump voters (notice the negative sign of the interaction term coefficient). This finding provides additional support for the hypothesized mechanism driving the divergence in social and political trust during a crisis. Specifically, dissatisfaction with the institutions and political forces expected to provide support leads to a decline in political trust and, hence, an increase in social trust. In essence, disillusioned citizens, particularly Trump voters in our context, play a pivotal role in steering this nuanced shift by actively seeking alternative sources of support within society.

Table S1. Within-sample correlation between Political Trust and Social trust (attrition weights)

	(1)	(2)	(3)
		Social Trust	
Trust Federal Government	0.0981***	0.0917**	0.101***
	(0.0367)	(0.0360)	(0.0367)
Trust Federal Government*Trump Voter	-0.0935*	-0.0898*	-0.0899*

	(0.0536)	(0.0532)	(0.0527)
Cumulative COVID-19 Deaths		-0.0266*	
		(0.0136)	
Direct COVID-19 Exposure			0.121***
			(0.0446)
Constant	0.0404***	0.0415***	0.0168
	(0.00671)	(0.00645)	(0.0105)
Observations	2,283	2,272	2,283
R-squared	0.008	0.010	0.016
Participants	973	966	973

Robust standard errors in parentheses; *** p<0.01, ** p<0.05, * p<0.1. The dependent variable is the result of principal component analysis of social trust, comprising three items for the evaluation of generalized social trust. Trust in the Federal Government is used as a independent variable. Dependent variables are standardized, coefficients represent outcome's change in terms of standard deviations.

7. Additional robustness checks

7.1 Post-stratification weights

A possible limit to our study concerns the external validity of the results, because the Amazon MTurk sample is non representative of the US population. To mitigate this concern, we repeat the empirical analysis using post-stratification weights, together with attrition weights to observe whether results change when we make our sample similar to the US population. Post-stratification weights are built comparing the distribution of gender, white people, black people, Asian people and college graduate in the sample to the one of the US population as in the American Community Survey (ACS). Tables A7-A14 replicate Tables 2-3 and R1-R3, R6, R8 and R9, Table A15 replicates Table 4, Table A16 replicates Table A17, while Figures A1, A4, A5 and A6 replicate Figures 1-4. Figures A3 and A8, replicate Figures A2 and A7 respectively.
Results and trends suffer negligible changes after the introduction of post-stratification weights, suggesting that our results might hold for the entire population. Generally speaking, including post-stratification weights does not change the main results: Trump voters' social trust grows, while trust in the Federal Government in the period considered falls. Experimental results tend to be robust to sample weighting as well. While the positive priming does not have an effect on Trump voters' trust in the federal government nor social trust, the effect of negative priming and results on respondents highly exposed to social media are indeed confirmed.

7.2 Using an alternative measure for being a Trump voters

A key finding of our study is the presence of partisan dynamics in social and trust in the Federal Government, with Trump voters driving the observed changes over time. However, the difference between Trump and non-Trump voters, rather than capturing the political leaning of respondents, might entail differences in socio-demographic characteristics between the two groups. These might, in turn, predict the observed changes in social and political trust. To mitigate this concern, we replicate our main estimates (Figure 2 and Table 3) replacing the 'Trump Voter' dummy with the residuals from a logistic regression, where the probability of being a Trump voter is used as dependent variable. This allows us to check whether the reduction (increase) of political (social) trust is confirmed when we use an alternative measure of political orientation, i.e. a measure that is 'cleaned' from the potential association between the (observed) socio-demographic characteristics with trust and the likelihood of voting for Trump. The residuals are derived from a model that predicts the probability of being Trump Voter using, as controls, age, gender, religion, ethnic group, working status, number of people in the household, number of diseases, and insurance status. Residuals should, therefore, represent ideological/political preferences in a much neater way than the 'Trump Voter' dummy. Regarding the interpretation, higher values of the unexplained probability of voting Trump (i.e. the residuals) imply stronger preference for Trump than what is predicted by socio-demographic factors. Results indeed confirm that trust in the Federal Government decreased while social trust increased

for individuals with higher residuals; at the same time, we do not find changes for other voters. This effect is significant only between the first and the second wave. Results are summarized in Figure A15 and A16 in the Appendix. Moreover, Figures A17 and A18 show that for people who are ideologically closer to Trump, the loss of institutional trust and the rise in social trust are associated to direct COVID-19 exposure. Overall, results in Figure 2 (Table R6) and Table 3 are robust to an alternative measure of being a Trump voter, which nets out the potential role of socio-demographic characteristics in explaining estimated partisan dynamics.

7.3 The effects of our stimuli on opinions and perceptions

Another potential concern refers to the validity of the stimuli used in the experiment. The validity of the experimental results hinges on the assumption that information treatments impacted on the respondent's perception in a direction that is consistent with the valence of the message. Hence, we verified whether the messages really yielded this effect through *ad hoc* manipulation checks. Consistent with the content of message received, respondents in the negative priming group were asked whether they think that the COVID-19 situation was better in the US or in the rest of the world and whether the US economy was doing better in April 2020 than in the last quarter of 2019. Respondents in the positive information condition received questions on whether the COVID-19 situation was better in November than in April and whether the economy was doing better than in April. The control group received all four questions. Manipulation checks confirm that our messages did affect the way respondents perceived the economic and COVID-19 situation. More positive evaluations were provided in the positive message condition than in the control condition. On the contrary, respondents in the negative message group were very critical. Results are summarized in Figure A19.

As an additional manipulation check, we measured the perceived valence of the stimulus, i.e. how much the piece of information received was perceived as positive (10) or negative (0). As shown in Figure A20, results confirm that respondents in the positive message group indeed perceived the statement as having a positive valence, while respondents in the negative message understood the pessimistic attitude of the message they read. However, both Figures A19 and A20 suggest that the positive message had a stronger influence than the negative one. In other words, the negative message was perceived as more negative than the positive message was perceived as positive.

Finally, we further explore the mechanisms driving our experimental results. An explanation to the partisan effects on trust might rest on partisan perceptions of the *valence* of the stimuli and on partisan *opinion changes* in response to such stimuli.

First, when looking at the impact of the stimuli on participants' beliefs, we do not find sizeable partisan differences. Relative to the control condition, Trump and non-Trump voters exposed to the message tended to report beliefs about the economic and health situation in line with the valence of the message received (Figure A21). Incidentally, this piece of evidence also suggests that our conditions were effective in changing opinions. A partisan difference seems to emerge, however, with respect to the magnitude of the *change in opinions*. Interpreting the differences in opinions between the message conditions and the control condition as within-person changes, the negative message generated larger changes in the beliefs of Trump voters than in those of non-Trump voters. The opposite occurs for the positive message, with larger opinion changes observed for non-Trump voters than for Trump voters. In other terms, with respect to the control condition, reading the negative (positive) message had a stronger (weaker) effect on the beliefs of Trump voters than on those of non-Trump voters. This can be explained, however, by more optimistic baseline opinions on the health and economic situation held by the former, as shown in the control condition.

Second, and consistent with the aforementioned findings for the entire sample, the manipulation checks by political affiliation highlight that the positive message was perceived as 'stronger' than the negative one *equally* by the Trump and by the non-Trump voters (Figure A22). The negative message was perceived, however, as being less negative by Trump voters, most likely because of partisan-motivated beliefs[13]. In other words, to reduce the distance between the message valence and their prior beliefs, Trump voters might have adjusted the perceived valence of the former in a way that is consistent with the latter.

Additional references

[1] Follmer, D. J., Sperling, R. A., & Suen, H. K. (2017). The role of MTurk in education research: Advantages, issues, and future directions. *Educational Researcher*, *46*(6), 329-334.

[2] Hauser, D. J., & Schwarz, N. (2016). Attentive Turkers: MTurk participants perform better on online attention checks than do subject pool participants. *Behavior research methods*, *48*, 400-407.

[3] Reeskens, T., & Hooghe, M. (2008). Cross-cultural measurement equivalence of generalized trust. Evidence from the European Social Survey (2002 and 2004). *Social indicators research*, *85*, 515-532.

[4] Banerjee, D., & Meena, K. S. (2021). COVID-19 as an "Infodemic" in Public Health: Critical Role of the Social Media. *Frontiers in Public Health*, *9*, 231.

[5] Nielsen, R. K., Fletcher, R., Newman, N., Brennen, J. S., & Howard, P. N. (2020). *Navigating the 'infodemic': How people in six countries access and rate news and information about coronavirus*. Reuters Institute.

[6] Alatas, V., Chandrasekhar, A. G., Mobius, M., Olken, B. A., & Paladines, C. (2019). *When celebrities speak: A nationwide Twitter experiment promoting vaccination in Indonesia* (No. w25589). National Bureau of Economic Research.

[7] Allcott, H., Boxell, L., Conway, J., Gentzkow, M., Thaler, M., & Yang, D. (2020a). Polarization and public health: Partisan differences in social distancing during the coronavirus pandemic. *Journal of Public Economics*, *191*, 104254.

[8] Allcott, H., Boxell, L., Conway, J., Gentzkow, M., Thaler, M., & Yang, D. (2020b). Polarization and public health: Partisan differences in social distancing during the coronavirus pandemic. *Journal of Public Economics*, *191*, 104254.

[9] Allcott, H., Braghieri, L., Eichmeyer, S., & Gentzkow, M. (2020c). The welfare effects of social media. *American Economic Review*, *110*(3), 629-76.

[10] Banerjee, A., Alsan, M., Breza, E., Chandrasekhar, A. G., Chowdhury, A., Duflo, E., ... & Olken, B. A. (2020). *Messages on COVID-19 prevention in India increased symptoms reporting and adherence to preventive behaviors among 25 million recipients with similar effects on non-recipient members of their communities* (No. w27496). National Bureau of Economic Research.

[11] Zhuravskaya, E., Petrova, M., & Enikolopov, R. (2020). Political effects of the internet and social media. *Annual Review of Economics*, *12*, 415-438.

[12] Conzo, P., Gallice, A., Morales, J. S., Samahita, M., & Taylor, L. K. (2021). *Can Hearts Change Minds? Social media Endorsements and Policy Preferences* (No. 641). Collegio Carlo Alberto.

[13] Bisgaard, M. (2015). Bias will find a way: Economic perceptions, attributions of blame, and partisan-motivated reasoning during crisis. *The journal of politics*, *77*(3), 849-860.

https://ideas.repec.org/p/cca/wpaper/641.html

Main regression tables

Table R1. Institutional trust dynamics

	(1) Institutional	(2) Trump	(3) WHO	(4) CDC	(5) FedGov	(6) LocGov	(7) Pharma	(8) SocMed	(9) Science	(10) News
Wave 2	-0.0958***	-0.0497*	-0.0503	-0.0487	-0.0627*	-0.0553	-0.0179	-0.0933**	-0.0690**	-0.120***
	(0.0332)	(0.0265)	(0.0324)	(0.0355)	(0.0360)	(0.0392)	(0.0345)	(0.0421)	(0.0333)	(0.0376)
Wave 3	-0.140***	-0.0703***	-0.0125	-0.103***	-0.139***	-0.166***	0.0155	-0.134***	0.0187	-0.193***
	(0.0282)	(0.0231)	(0.0314)	(0.0367)	(0.0312)	(0.0345)	(0.0335)	(0.0381)	(0.0293)	(0.0329)
Constant	0.0863***	0.0757***	0.0176	0.0196	0.0882***	0.0671***	0.0636***	0.130***	-0.0384***	0.0874***
	(0.0148)	(0.0117)	(0.0150)	(0.0164)	(0.0159)	(0.0177)	(0.0158)	(0.0190)	(0.0148)	(0.0169)
Observations	2,283	2,283	2,283	2,283	2,283	2,283	2,283	2,283	2,283	2,283
R-squared	0.019	0.007	0.003	0.006	0.013	0.015	0.001	0.010	0.007	0.025
Participants	973	973	973	973	973	973	973	973	973	973
Test Wave 3-Wave 2=0	-0.0442	-0.0207	0.0378	-0.0544	-0.0762**	-0.111***	0.0334	-0.0405	0.0877***	-0.0728**
	(0.0299)	(0.0253)	(0.0314)	(0.0381)	(0.0338)	(0.0355)	(0.0349)	(0.0399)	(0.0314)	(0.0345)

Robust standard errors in parentheses; *** p<0.01, ** p<0.05, * p<0.1. Column (1) uses as a dependent variable the result of principal component analysis comprising all other institutional trust variables. Column (2)-(10) use as dependent variable trust in a specific institution indicated by the column heading. Dependent variables are standardized, coefficients represent outcome's change in terms of standard deviations.

Table R2. Dynamics of attribution of responsibility

	(1) Chinese Government	(2) US Government	(3) WHO	(4) CDC	(5) Trump	(6) Chinese Public	(7) US Public
Wave 2	-0.0557*	-0.0569*	-0.0623**	-0.115***	-0.0161	-0.162***	-0.0848**
	(0.0309)	(0.0309)	(0.0281)	(0.0382)	(0.0338)	(0.0325)	(0.0348)
Wave 3	-0.251***	0.151***	0.140***	-0.0424	-0.138***	-0.212***	0.373***
	(0.0366)	(0.0375)	(0.0316)	(0.0411)	(0.0386)	(0.0356)	(0.0399)
Constant	0.0965***	0.00742	0.00193	0.100***	0.0806***	0.165***	-0.0141
	(0.0148)	(0.0151)	(0.0134)	(0.0180)	(0.0163)	(0.0154)	(0.0167)
Observations	2,281	2,281	2,278	2,281	2,281	2,280	2,280
R-squared	0.038	0.024	0.029	0.008	0.010	0.035	0.093
Participants	973	973	972	973	973	973	973
Test Wave 3 – Wave 2=0	-0.195***	0.208***	0.202***	0.0721*	-0.122***	-0.0496	0.457***
	(0.0375)	(0.0374)	(0.0321)	(0.0418)	(0.0384)	(0.0358)	(0.0401)

Robust standard errors in parentheses; *** p<0.01, ** p<0.05, * p<0.1. Dependent variables represent to what extent respondents attribute responsibility for the current COVID-19 situation in the US to the actor indicated in the column heading. Dependent variables are standardized, coefficients represent outcome's change in terms of standard deviations.

Table R3. Dynamics of social trust

	(1)<br>Social	(2)<br>Most People can be Trusted	(3)<br>Most People Fair	(4)<br>Most People Helpful
Wave 2	0.0456**	0.0331	0.0610*	0.0299
	(0.0227)	(0.0249)	(0.0328)	(0.0322)
Wave 3	-0.0159	-0.0510**	0.00806	0.000313
	(0.0236)	(0.0249)	(0.0319)	(0.0331)
Constant	0.0197*	0.0402***	-0.00207	0.0145
	(0.0104)	(0.0113)	(0.0152)	(0.0147)
Observations	2,283	2,283	2,283	2,283
R-squared	0.006	0.008	0.004	0.001
Participants	973	973	973	973
Test Wave 3 – Wave 2=0	-0.0615**	-0.0841***	-0.0530*	-0.0296
	(0.0253)	(0.0266)	(0.0320)	(0.0353)

Robust standard errors in parentheses; *** p<0.01, ** p<0.05, * p<0.1. The dependent variable in column (1) is a principal component analysis variable that is based on three items, which are used as dependent variables in column (2)-(4). Column (2) indicates the extent respondents believe "most people can be trusted" vs "you can not be too careful". Column (3) indicates to what extent "most people would try to take advantage of me" vs "most people would try to be fair" according to the respondent. Column (4) represents how much respondents believe that "people mostly look out for themselves" vs "people mostly try to be helpful". Dependent variables are standardized, coefficients represent outcome's change in terms of standard deviations.

Table R4. Dynamics of social trust and Institutional Trust

	(1)	(2)	(3)	(4)
	Institutional Trust		Social Trust	
Wave 2	-0.104***	-0.0932***	0.0621***	0.0426*
	(0.0346)	(0.0330)	(0.0238)	(0.0225)
Wave 3	-0.173***	-0.118***	0.0393	-0.0415*
	(0.0432)	(0.0316)	(0.0342)	(0.0251)
Cumulative COVID-19 Deaths	0.0267		-0.0422**	
	(0.0258)		(0.0199)	
Direct COVID-19 Exposure		-0.121*		0.143***
		(0.0662)		(0.0476)
Constant	0.0955***	0.104***	0.00311	-0.00109
	(0.0188)	(0.0172)	(0.0137)	(0.0128)
Observations	2,272	2,283	2,272	2,283
R-squared	0.019	0.023	0.009	0.015
Participants	966	973	966	973
Test Wave 3 – Wave 2=0	-0.0685*	-0.0253	-0.0228	-0.0841***
	(0.0378)	(0.0326)	(0.0305)	(0.0272)

Robust standard errors in parentheses; *** p<0.01, ** p<0.05, * p<0.1. Columns (1)-(2) use a pca institutional trust variable, including trust in all institutional targets, as a dependent variable, while columns (3)-(4) use a pca variable of social trust, comprising three items for the evaluation of generalized social trust. Dependent variables are standardized, coefficients represent outcome's change in terms of standard deviations.

Table R5. Dynamics of social trust and institutional trust by exposure to COVID and political orientation

	(1)	(2)	(3)	(4)
	Institutional Trust		Social Trust	
Wave 2	-0.103***	-0.0944***	0.0619***	0.0431*
	(0.0346)	(0.0328)	(0.0238)	(0.0225)
Wave 3	-0.169***	-0.128***	0.0387	-0.0374
	(0.0429)	(0.0310)	(0.0343)	(0.0252)
Direct COVID-19 Exposure		0.0292		0.0761
		(0.0568)		(0.0562)
Direct COVID-19 Exposure*Trump. Voter		-0.351***		0.157*
		(0.130)		(0.0926)
Cumulative COVID-19 Deaths	0.0548**		-0.0469**	
	(0.0237)		(0.0221)	
Cumulative COVID-19 Deaths*Trump Voter	-0.107**		0.0177	
	(0.0444)		(0.0289)	
Constant	0.0898***	0.115***	0.00406	-0.00602
	(0.0189)	(0.0184)	(0.0137)	(0.0134)
Observations	2,272	2,283	2,272	2,283
R-squared	0.024	0.031	0.009	0.018
Participants	966	973	966	973
Test Wave 3 – Wave 2=0	-0.0656*	-0.0331	-0.0233	-0.0805***
	(0.0378)	(0.0319)	(0.0305)	(0.0272)

Robust standard errors in parentheses; *** p<0.01, ** p<0.05, * p<0.1. Columns (1)-(2) use a pca institutional trust variable, including trust in all institutional targets, as a dependent variable, while columns (3)-(4) use a pca variable of social trust, comprising three items for the evaluation of generalized social trust. Dependent variables are standardized, coefficients represent outcome's change in terms of standard deviations.

Table R6. Partisan trust dynamics

	(1)	(2)	(3)	(4)	(5)	(6)
	Trust in The Federal Government			Social Trust		
Wave 2	0.0116	-0.00346	0.0143	0.00700	0.0258	0.00347
	(0.0313)	(0.0333)	(0.0313)	(0.0256)	(0.0265)	(0.0254)
Wave 3	-0.0606*	-0.121**	-0.0408	-0.0568**	-0.00227	-0.0833***
	(0.0326)	(0.0487)	(0.0356)	(0.0281)	(0.0367)	(0.0298)
Wave 2*Trump voter	-0.211**	-0.211**	-0.212**	0.110**	0.101**	0.111**
	(0.0893)	(0.0898)	(0.0888)	(0.0507)	(0.0506)	(0.0503)
Wave 3*Trump voter	-0.246***	-0.244***	-0.247***	0.129**	0.121**	0.131***
	(0.0746)	(0.0747)	(0.0747)	(0.0509)	(0.0507)	(0.0504)
Cumulative COVID-19 Deaths		0.0484*			-0.0397**	
		(0.0278)			(0.0201)	
Direct COVID-19 Exposure			-0.108			0.145***
			(0.0727)			(0.0471)
Constant	0.0872***	0.105***	0.103***	0.0202**	0.00470	-0.000756
	(0.0156)	(0.0198)	(0.0187)	(0.0103)	(0.0136)	(0.0127)
Observations	2,283	2,272	2,283	2,283	2,272	2,283
R-squared	0.025	0.028	0.028	0.013	0.015	0.022
Participants	973	966	973	973	966	973
Test Wave 2 – Wave 3=0	-0.0723**	-0.117***	-0.0550	-0.0638**	-0.0280	-0.0868***
	(0.0354)	(0.0430)	(0.0375)	(0.0294)	(0.0330)	(0.0312)
Test (Wave 2 – Wave 3)*Trump=0	-0.0345	-0.0331	-0.0352	0.0186	0.0198	0.0195
	(0.0807)	(0.0810)	(0.0808)	(0.0562)	(0.0562)	(0.0558)

Robust standard errors in parentheses; *** p<0.01, ** p<0.05, * p<0.1. Columns (1)-(2)-(3) use trust in the Federal Government, as a dependent variable, while columns (4)-(5)-(6) use a pca variable of social trust, comprising three items for the evaluation of generalized social trust. Dependent variables are standardized, coefficients represent outcome's change in terms of standard deviations.

Table R7. Dynamics of social trust and institutional trust by partisanship

	(1)	(2)	(3)	(4)	(5)	(6)
		Institutional Trust			Social Trust	
Wave 2	0.00420	-0.00253	0.00721	0.00700	0.0258	0.00347
	(0.0263)	(0.0276)	(0.0263)	(0.0256)	(0.0265)	(0.0254)
Wave 3	-0.0694**	-0.0942**	-0.0467	-0.0568**	-0.00227	-0.0833***
	(0.0283)	(0.0423)	(0.0312)	(0.0281)	(0.0367)	(0.0298)
Wave 2*Trump voter	-0.285***	-0.283***	-0.286***	0.110**	0.101**	0.111**
	(0.0840)	(0.0844)	(0.0832)	(0.0507)	(0.0506)	(0.0503)
Wave 3*Trump voter	-0.214***	-0.214***	-0.216***	0.129**	0.121**	0.131***
	(0.0692)	(0.0694)	(0.0691)	(0.0509)	(0.0507)	(0.0504)
Cumulative COVID-19 Deaths		0.0202			-0.0397**	
		(0.0256)			(0.0201)	
Direct COVID-19 Exposure			-0.124*			0.145***
			(0.0651)			(0.0471)
Constant	0.0849***	0.0913***	0.103***	0.0202**	0.00470	-0.000756
	(0.0143)	(0.0180)	(0.0165)	(0.0103)	(0.0136)	(0.0127)
Observations	2,283	2,272	2,283	2,283	2,272	2,283
R-squared	0.040	0.040	0.044	0.013	0.015	0.022
Participants	973	966	973	973	966	973
Test Wave 3 – Wave 2=0	-0.0736**	-0.0917**	-0.0539*	-0.0638**	-0.0280	-0.0868***
	0.0294	0.0373	0.0316	0.0294	0.0330	0.0312
Test (Wave 3 – Wave 2)*Trump=0	0.0701	0.0696	0.0693	0.0186	0.0198	0.0195
	(0.0740)	(0.0742)	(0.0740)	(0.0562)	(0.0562)	(0.0558)

Robust standard errors in parentheses; *** p<0.01, ** p<0.05, * p<0.1. Columns (1)-(2)-(3) use a pca institutional trust variable, including trust in all institutional targets, as a dependent variable, while columns (4)-(5)-(6) use a pca variable of social trust, comprising three items for the evaluation of generalized social trust. Dependent variables are standardized, coefficients represent outcome's change in terms of standard deviations.

Table R8. Partisan dynamics of institutional trust

	(1) Institutions	(2) Trump	(3) WHO	(4) CDCs	(5) FedGov	(6) LocGov	(7) Pharma	(8) SocMed	(9) Science	(10) News
Wave 2	0.00420	0.00390	0.0388	0.0219	0.0116	-0.000911	0.0571*	-0.0882**	0.0313	-0.0660**
	(0.0263)	(0.0204)	(0.0312)	(0.0376)	(0.0313)	(0.0376)	(0.0344)	(0.0394)	(0.0320)	(0.0317)
Wave 3	-0.0694**	-0.0508**	0.0512	-0.0578	-0.0606*	-0.155***	0.0235	-0.109**	0.101***	-0.119***
	(0.0283)	(0.0227)	(0.0344)	(0.0413)	(0.0326)	(0.0361)	(0.0364)	(0.0438)	(0.0303)	(0.0346)
Wave 2*Trump voter	-0.285***	-0.152**	-0.253***	-0.201**	-0.211**	-0.155	-0.214***	-0.0146	-0.285***	-0.155
	(0.0840)	(0.0680)	(0.0775)	(0.0821)	(0.0893)	(0.0950)	(0.0815)	(0.103)	(0.0790)	(0.0946)
Wave 3*Trump voter	-0.214***	-0.0533	-0.193***	-0.136	-0.246***	-0.0234	-0.00938	-0.0834	-0.255***	-0.235***
	(0.0692)	(0.0583)	(0.0733)	(0.0848)	(0.0746)	(0.0845)	(0.0797)	(0.0844)	(0.0711)	(0.0786)
Constant	0.0849***	0.0749***	0.0164	0.0187	0.0872***	0.0664***	0.0626***	0.129***	-0.0398***	0.0866***
	(0.0143)	(0.0115)	(0.0147)	(0.0163)	(0.0156)	(0.0175)	(0.0155)	(0.0188)	(0.0145)	(0.0165)
Observations	2,283	2,283	2,283	2,283	2,283	2,283	2,283	2,283	2,283	2,283
R-squared	0.040	0.016	0.018	0.013	0.025	0.019	0.011	0.011	0.029	0.034
Participants	973	973	973	973	973	973	973	973	973	973
Test Wave 3 - Wave 2=0	-0.0736**	-0.0547**	0.0124	-0.0797*	-0.0723**	-0.154***	-0.0336	-0.0204	0.0702**	-0.0535
	(0.0294)	(0.0249)	(0.0344)	(0.0429)	(0.0354)	(0.0362)	(0.0376)	(0.0448)	(0.0323)	(0.0354)
Test (Wave 3 – Wave 2)*Trump=0	0.0701	0.0991	0.0600	0.0649	-0.0345	0.132	0.204**	-0.0688	0.0303	-0.0804
	(0.0740)	(0.0629)	(0.0730)	(0.0879)	(0.0807)	(0.0876)	(0.0823)	(0.0901)	(0.0764)	(0.0834)

Robust standard errors in parentheses; *** p<0.01, ** p<0.05, * p<0.1. Column (1) uses as a dependent variable the result of principal component analysis comprising all other institutional trust variables. Column (2)-(10) use as dependent variable trust in a specific institution indicated by the column heading. Dependent variables are standardized, coefficients represent outcome's change in terms of standard deviations.

Table R9. Partisan dynamics of attribution of responsibilities

	(1) Chinese Government	(2) US Government	(3) WHO	(4) CDC	(5) Trump	(6) Chinese Public	(7) US Public
Wave 2	-0.0536	-0.0368	-0.0489	-0.160***	-0.103***	-0.177***	-0.0668
	(0.0340)	(0.0372)	(0.0338)	(0.0463)	(0.0380)	(0.0391)	(0.0438)
Wave 3	-0.320***	0.232***	0.180***	-0.0677	-0.184***	-0.239***	0.461***
	(0.0434)	(0.0448)	(0.0384)	(0.0485)	(0.0431)	(0.0399)	(0.0476)
Wave 2*Trump voter	-0.00647	-0.0568	-0.0380	0.129	0.247***	0.0421	-0.0505
	(0.0704)	(0.0664)	(0.0604)	(0.0811)	(0.0750)	(0.0699)	(0.0722)
Wave 3*Trump voter	0.233***	-0.265***	-0.133**	0.0742	0.133	0.0864	-0.291***
	(0.0780)	(0.0798)	(0.0667)	(0.0916)	(0.0901)	(0.0840)	(0.0856)
Constant	0.0965***	0.00709	0.00172	0.101***	0.0819***	0.165***	-0.0144
	(0.0147)	(0.0150)	(0.0133)	(0.0179)	(0.0161)	(0.0154)	(0.0167)
Observations	2,281	2,281	2,278	2,281	2,281	2,280	2,280
R-squared	0.046	0.033	0.032	0.010	0.021	0.036	0.101
Participants	973	973	972	973	973	973	973
Test Wave 3 – Wave 2=0	-0.267***	0.269***	0.229***	0.0922*	-0.0803*	-0.0616	0.528***
	(0.0453)	(0.0453)	(0.0390)	(0.0489)	(0.0439)	(0.0401)	(0.0494)
Test (Wave 3 – Wave 2)*Trump=0	0.239***	-0.208***	-0.0945	-0.0545	-0.114	0.0443	-0.241***
	(0.0788)	(0.0794)	(0.0684)	(0.0936)	(0.0880)	(0.0843)	(0.0839)

Robust standard errors in parentheses; *** p<0.01, ** p<0.05, * p<0.1. Dependent variables represent to what extent respondents attribute responsibility for the current COVID-19 situation in the US to the actor indicated in the column heading. Dependent variables are standardized, coefficients represent outcome's change in terms of standard deviations.

Additional tables

Table A1. Description of variables.

Variable	Description
Institutional trust	Result of principal component analysis on all institutional trust variables: trust in Trump, the WHO, CDCs, federal government, local government, pharmaceutical companies, social media, science and news.
Social trust	Result of principal component analysis on Item 1-3.
Trust in Trump, the WHO (World Health Organization), CDCs (Centres for Disease Control and Prevention), Federal Government, local government, pharmaceutical companies, social media, science and news	Asked through the following question "How much would you say you trust the following…(institution name)?" Answers are scaled from 1 (Not at all) to 5 (A great deal).
Item 1 (Social trust)	Answer to the question: "Generally speaking, would you say that most people can be trusted, or that you can't be too careful in dealing with people?", where 5 (maximum value) represents "Most people can be trusted".
Item 2 (Social trust)	Answer to the question: "Do you think that most people would try to take advantage of you if they got the chance, or would they try to be fair?", where 5 represents "Most people would try to be fair".
Item 3 (Social trust)	Answer to the question: "Would you say that most of the time people try to be helpful or that they are mostly looking out for themselves?, where 5 is "People mostly try to be helpful".
Attribution of responsibility to Chinese Government, US Government, the WHO, CDCs, Trump, Chinese public and US public.	Answer to the question: "To what extent do you think each of the following is responsible for the current situation of COVID-19 in the US?". Answers are scaled from 0 to 100, with 100 being "Most responsible". To the purpose of this research answers are rescaled from 0 to 10.
Trump voter indicator	It is one (1) if the respondent voted for Trump at the 2016 elections and zero (0) otherwise. It is zero if respondents voted either for Clinton or Other.
Direct COVID-19 Exposure	It is an indicator variable which is one (1) if either the respondent, one of their friends or members of immediate or extended family, has been diagnosed with COVID-19. It is zero (0) otherwise.
Cumulative COVID-19 Deaths	Measure indirect exposure to COVID-19. It is the relative number of deaths in the county of the respondent since the pandemic started, expressed in percentage.

Table A2. Descriptive statistics, socio-demographic characteristics.

Gender	%
Male	56.98
Female	42.71
Other	0.31

Direct Exposure	%
Yes	13.38
No	86.62

Voting 2016	%
Trump	36.04
Clinton	41.07
Other	22.90

Race	%
White	71.66
Hispanic	9.24
Black	11.91
Asian	6.37
Other	0.82

Education	%
Less than high school	0.10
High school	10.37
Some college	15.91
Associate degree	10.99
Bachelor's degree	48.05
Master	12.22
PhD	1.03
JD MD	1.33

Table A3. Descriptive statistics, continuous variables

Variable	Mean (Std. Err.)	Min	Max
Age	38.328 (11.284)	19	75
Cumulative COVID-19 Deaths	0.0265 (0.0465)	0	0.3519
Institutional Trust (pca)	0.0000 (0.0399)	-4.5745	6.5152
Trust Trump	2.0201 (1.2293)	1	5
Trust WHO	3.0232 (1.2319)	1	5
Trust CDCs	3.2240 (1.1162)	1	5
Trust federal government	2.1063 (0.9419)	1	5
Trust local government	2.6474 (1.0388)	1	5
Trust pharmaceutical companies	2.1382 (1.0377)	1	5
Trust social media	2.0687 (0.9439)	1	5
Trust science	3.9103 (1.0910)	1	5
Trust news	2.7528 (1.0071)	1	5
Social Trust (pca)	0.0000 (1.5683)	-0.4470	0.1445
Item 1	3.1629 (1.1817)	1	5
Item 2	3.3115 (1.1042)	1	5
Item 3	3.2117 (1.1785)	1	5
Attribution to Chinese Gov.	5.8607 (3.2430)	0	10
Attribution to US Gov.	5.5656 (3.1543)	0	10
Attribution to WHO	5.6812 (3.5519)	0	10
Attribution to CDCs	3.4939 (2.9472)	0	10
Attribution to Trump	4.0060 (3.2632)	0	10
Attribution to Chinese Pub.	3.3639 (3.3341)	0	10
Attribution to US Pub.	4.2090 (3.1181)	0	10

Table A4. Attrition

Attrition	N°	%
Present in all waves	504	51.75
Only wave 1 and 2	215	22.07
Only wave 1	166	17.04
Only wave 1 and 3	89	9.14

Table A5. Attrition logit Model

	Probability of Attrition
Age	-0.000937
	(0.00373)
Female	-0.0350
	(0.0726)
White	0.127
	(0.185)
Black	0.127
	(0.193)
Asian	0.237
	(0.238)
Hispanic	-0.258***
	(0.0937)
Catholic	0.182
	(0.134)
Protestant	0.0197
	(0.140)
Not religious	-0.169
	(0.130)
Graduate	0.0150
	(0.0881)
Married	0.174*
	(0.0923)
Divorced	0.0294
	(0.214)
Separated	-0.481
	(0.648)
Cohabiting	0.159
	(0.138)
Insured	0.0661
	(0.0746)
Voted Trump	-0.103
	(0.0810)
N° Diseases	-0.0929**
	(0.0438)
Income	0.0553
	(0.0358)
Missing Income	-2.505***
	(0.236)
Social Trust before attrition	-0.00301
	(0.0280)
Institutional Trust before attrition	-0.0173
	(0.0173)
Constant	2.514***
	(0.348)
Observations	2,919

Robust standard errors in parentheses
*** p<0.01, ** p<0.05, * p<01

Table A6. Dynamics of being exposed to COVID-19.

	(1)	(2)
	Fixed-effects Linear Probability Model	
	Diagnosed Self	Direct Exposure
Wave 2	-0.000750	0.0155
	(0.00813)	(0.0119)
Wave 3	0.00775	0.157***
	(0.0107)	(0.0332)
Wave 2*Trump voter	-0.00267	-0.00526
	(0.0218)	(0.0330)
Wave 3*Trump voter	0.0171	-0.00948
	(0.0183)	(0.0416)
Containment policy	0.000947*	7.94e-05
	(0.000497)	(0.00145)
Protective behavior	-0.00279	0.00829
	(0.00636)	(0.0104)
Cumulative COVID-19 Deaths	0.168	0.554
	(0.122)	(0.347)
Constant	0.00827	0.135
	(0.0292)	(0.0843)
Observations	2,239	2,271
R-squared	0.009	0.092
Number of participants	962	965
Test Wave 3-Wave 2=0	0.00850	0.141***
	(0.0105)	(0.0314)
Test (Wave 3-Wave 2)*Trump=0	0.0198	-0.00422
	(0.0200)	(0.0436)

Robust standard errors in parentheses; *** p<0.01, ** p<0.05, * p<0.1. The dependent variable in columns (1)-(2) are indicators of whether the respondent has been diagnosed with COVID-19 or not, and whether the respondent or one of their loved ones (relatives or friends) has been diagnosed with COVID-19, respectively. Regressions control for the strength of containment policies, measured by the Oxford COVID-19 policy response tracker, the extent respondents complied with forms of protective behavior - including work from home, washing hands, buying masks and social distancing – and cumulative COVID-19 deaths in the county of the respondent.

Table A7. Model Table R1 with post-stratification weights

	(1) Institutional	(2) Trump	(3) WHO	(4) CDC	(5) FedGov	(6) LocGov	(7) Pharma	(8) SocMed	(9) Science	(10) News
Wave 2	-0.0840**	-0.0362	-0.0540	-0.0507	-0.0372	-0.0489	0.0186	-0.124***	-0.0526*	-0.117***
	(0.0340)	(0.0234)	(0.0366)	(0.0422)	(0.0343)	(0.0396)	(0.0383)	(0.0417)	(0.0319)	(0.0366)
Wave 3	-0.121***	-0.0391*	0.0125	-0.0931**	-0.122***	-0.151***	0.0484	-0.165***	-0.00619	-0.170***
	(0.0329)	(0.0223)	(0.0365)	(0.0425)	(0.0360)	(0.0398)	(0.0397)	(0.0470)	(0.0330)	(0.0379)
Constant	-0.0373**	-0.0190*	-0.0365**	-0.00219	-0.0530***	-0.0248	-0.0868***	0.0677***	-0.0249	-0.0143
	(0.0161)	(0.0108)	(0.0176)	(0.0198)	(0.0165)	(0.0189)	(0.0188)	(0.0204)	(0.0152)	(0.0180)
Observations	2,283	2,283	2,283	2,283	2,283	2,283	2,283	2,283	2,283	2,283
R-squared	0.016	0.004	0.004	0.005	0.012	0.015	0.002	0.018	0.003	0.024
Participants	973	973	973	973	973	973	973	973	973	973
Test Wave 3 – Wave 2=0	-0.0374	-0.00293	0.0665*	-0.0423	-0.0847**	-0.103**	0.0297	-0.0410	0.0464	-0.0528
	(0.0338)	(0.0243)	(0.0368)	(0.0460)	(0.0370)	(0.0413)	(0.0385)	(0.0487)	(0.0345)	(0.0364)

Robust standard errors in parentheses; *** p<0.01, ** p<0.05, * p<0.1. Column (1) uses as a dependent variable the result of principal component analysis comprising all other institutional trust variables. Column (2)-(10) use as dependent variable trust in a specific institution indicated by the column heading. Dependent variables are standardized, coefficients represent outcome's change in terms of standard deviations.

Table A8. Model Table R2 with post-stratification weights

	(1) Chinese Government	(2) US Government	(3) WHO	(4) CDC	(5) Trump	(6) Chinese Public	(7) US Public
Wave 2	-0.0438	-0.0601*	-0.0484	-0.158***	-0.0255	-0.197***	-0.0997**
	(0.0346)	(0.0358)	(0.0352)	(0.0489)	(0.0458)	(0.0365)	(0.0403)
Wave 3	-0.261***	0.170***	0.144***	-0.0659	-0.183***	-0.246***	0.378***
	(0.0434)	(0.0473)	(0.0389)	(0.0510)	(0.0460)	(0.0435)	(0.0486)
Constant	0.115***	-0.0166	-0.0234	0.0363	0.0611***	0.103***	-0.0896***
	(0.0176)	(0.0185)	(0.0171)	(0.0241)	(0.0221)	(0.0184)	(0.0203)
Observations	2,281	2,281	2,278	2,281	2,281	2,280	2,280
R-squared	0.045	0.030	0.027	0.014	0.019	0.049	0.105
Participants	973	973	972	973	973	973	973
Test Wave 3 - Wave 2=0	-0.217***	0.230***	0.192***	0.0916*	-0.158***	-0.0490	0.478***
	(0.0443)	(0.0483)	(0.0409)	(0.0490)	(0.0456)	(0.0437)	(0.0490)

Robust standard errors in parentheses; *** p<0.01, ** p<0.05, * p<0.1. Dependent variables represent to what extent respondents attribute responsibility for the current COVID-19 situation in the US to the actor indicated in the column heading. Dependent variables are standardized, coefficients represent outcome's change in terms of standard deviations.

Table A9. Model Table R3 with post-stratification weights

	(1)	(2)	(3)	(4)
	Social	Most People can be Trusted	Most People Fair	Most People Helpful
Wave 2	0.0167	0.0137	0.0240	0.00759
	(0.0242)	(0.0263)	(0.0358)	(0.0364)
Wave 3	-0.0207	-0.0554*	-0.00453	0.00406
	(0.0297)	(0.0308)	(0.0389)	(0.0415)
Constant	-0.0707***	-0.0727***	-0.0772***	-0.0425**
	(0.0123)	(0.0132)	(0.0179)	(0.0179)
Observations	2,283	2,283	2,283	2,283
R-squared	0.002	0.006	0.001	0.000
Participants	973	973	973	973
Test Wave 3 – Wave 2=0	-0.0374	-0.0690**	-0.0285	-0.00352
	(0.0297)	(0.0307)	(0.0368)	(0.0427)

Robust standard errors in parentheses; *** p<0.01, ** p<0.05, * p<0.1. The dependent variable in column (1) is a principal component analysis variable that is based on three items, which are used as dependent variables in column (2)-(4). Column (2) indicates the extent respondents believe "most people can be trusted" vs "you can not be too careful". Column (3) indicates to what extent "most people would try to take advantage of me" vs "most people would try to be fair" according to the respondent. Column (4) represents how much respondents believe that "people mostly look out for themselves" vs "people mostly try to be helpful". Dependent variables are standardized, coefficients represent outcome's change in terms of standard deviations.

Table A10 Model Table 2 with post-stratification weights

	(1)	(2)	(3)	(4)
	Trust in The Federal Government		Social Trust	
Wave 2	-0.0480	-0.0367	0.0241	0.0143
	(0.0354)	(0.0344)	(0.0260)	(0.0239)
Wave 3	-0.165***	-0.117***	0.00414	-0.0441
	(0.0518)	(0.0401)	(0.0419)	(0.0315)
Cumulative COVID-19 Deaths	0.0353		-0.0194	
	(0.0288)		(0.0236)	
Direct COVID-19 Exposure		-0.0276		0.123**
		(0.0701)		(0.0589)
Constant	-0.0400*	-0.0499***	-0.0787***	-0.0845***
	(0.0211)	(0.0176)	(0.0167)	(0.0144)
Observations	2,272	2,283	2,272	2,283
R-squared	0.013	0.012	0.003	0.009
Participants	966	973	966	973
Wave 3 – Wave 2=0	-0.117**	-0.0800**	-0.0200	-0.0584*
	(0.0468)	(0.0399)	(0.0358)	(0.0324)

Robust standard errors in parentheses; *** p<0.01, ** p<0.05, * p<0.1. Columns (1)-(2) use trust in the Federal Governenment, as a dependent variable, while columns (3)-(4) use a pca variable of social trust, comprising three items for the evaluation of generalized social trust. Dependent variables are standardized, coefficients represent outcome's change in terms of standard deviations.

Table A11 Model Table R6 with post-stratification weights

	(1)	(2)	(3)	(4)	(5)	(6)
	Trust in The Federal Government			Social Trust		
Wave 2	0.0459	0.0362	0.0466	-0.0184	-0.0102	-0.0212
	(0.0359)	(0.0370)	(0.0359)	(0.0286)	(0.0306)	(0.0283)
Wave 3	-0.0582	-0.0983*	-0.0518	-0.0556	-0.0311	-0.0806**
	(0.0401)	(0.0522)	(0.0431)	(0.0357)	(0.0470)	(0.0378)
Wave 2*Trump voter	-0.272***	-0.273***	-0.272***	0.115**	0.111**	0.116**
	(0.0810)	(0.0814)	(0.0810)	(0.0527)	(0.0528)	(0.0523)
Wave 3*Trump voter	-0.216**	-0.216**	-0.217***	0.119*	0.116*	0.123**
	(0.0842)	(0.0843)	(0.0841)	(0.0634)	(0.0633)	(0.0627)
Cumulative COVID-19 Deaths		0.0331			-0.0184	
		(0.0271)			(0.0245)	
Direct COVID-19 Exposure			-0.0320			0.126**
			(0.0698)			(0.0582)
Constant	-0.0535***	-0.0415**	-0.0500***	-0.0705***	-0.0781***	-0.0846***
	(0.0161)	(0.0205)	(0.0172)	(0.0122)	(0.0167)	(0.0142)
Observations	2,283	2,272	2,283	2,283	2,272	2,283
R-squared	0.030	0.031	0.030	0.008	0.009	0.016
Participants	973	966	973	973	966	973
Test Wave 3 – Wave 2=0	-0.104**	-0.134***	-0.0984**	-0.0371	-0.0209	-0.0594
	(0.0417)	(0.0481)	(0.0437)	(0.0348)	(0.0399)	(0.0370)
Test (Wave 3 – Wave 2)*Trump=0	0.0561	0.0573	0.0554	0.00420	0.00494	0.00710
	(0.0859)	(0.0860)	(0.0859)	(0.0665)	(0.0664)	(0.0661)

Robust standard errors in parentheses; *** p<0.01, ** p<0.05, * p<0.1. Columns (1)-(2) use trust in the Federal Government, as a dependent variable, while columns (3)-(4) use a pca variable of social trust, comprising three items for the evaluation of generalized social trust. Dependent variables are standardized, coefficients represent outcome's change in terms of standard deviations.

Table A12 Model Table R8 with post-stratification weights

	(1)	(2)	(3)	(4)	(5)	(6)	(7)	(8)	(9)	(10)
	Institutions	Trump	WHO	CDCs	FedGov	LocGov	Pharma	SocMed	Science	News
Wave 2	0.0275	0.0136	0.0585	0.0389	0.0459	0.0142	0.0984**	-0.103**	0.0165	-0.0569
	(0.0353)	(0.0208)	(0.0415)	(0.0489)	(0.0359)	(0.0450)	(0.0456)	(0.0470)	(0.0370)	(0.0379)
Wave 3	-0.0628*	-0.0208	0.0676*	-0.0398	-0.0582	-0.148***	0.0334	-0.139**	0.0553	-0.0992**
	(0.0364)	(0.0213)	(0.0393)	(0.0478)	(0.0401)	(0.0462)	(0.0449)	(0.0575)	(0.0356)	(0.0407)
Wave 2*Trump voter	-0.365***	-0.163***	-0.368***	-0.293***	-0.272***	-0.206**	-0.261***	-0.0691	-0.226***	-0.196**
	(0.0791)	(0.0626)	(0.0799)	(0.0925)	(0.0810)	(0.0895)	(0.0814)	(0.0969)	(0.0709)	(0.0897)
Wave 3*Trump voter	-0.195**	-0.0595	-0.182**	-0.178*	-0.216**	-0.00568	0.0614	-0.0889	-0.209**	-0.242***
	(0.0778)	(0.0600)	(0.0887)	(0.0993)	(0.0842)	(0.0903)	(0.0918)	(0.0981)	(0.0815)	(0.0926)
Constant	-0.0380**	-0.0193*	-0.0372**	-0.00277	-0.0535***	-0.0252	-0.0874***	0.0676***	-0.0253*	-0.0147
	(0.0156)	(0.0106)	(0.0171)	(0.0195)	(0.0161)	(0.0187)	(0.0186)	(0.0204)	(0.0151)	(0.0178)
Observations	2,283	2,283	2,283	2,283	2,283	2,283	2,283	2,283	2,283	2,283
R-squared	0.050	0.015	0.033	0.019	0.030	0.023	0.019	0.019	0.018	0.036
Participants	973	973	973	973	973	973	973	973	973	973
Test Wave 3 – Wave 2=0	-0.0903**	-0.0344	0.00917	-0.0786	-0.104**	-0.162***	-0.0650	-0.0363	0.0388	-0.0423
	(0.0372)	(0.0246)	(0.0435)	(0.0537)	(0.0417)	(0.0466)	(0.0439)	(0.0595)	(0.0374)	(0.0391)
Test (Wave 3 – Wave 2)*Trump=0	0.170**	0.103*	0.186**	0.115	0.0561	0.200**	0.322***	-0.0198	0.0167	-0.0457
	(0.0791)	(0.0627)	(0.0799)	(0.103)	(0.0859)	(0.0952)	(0.0851)	(0.102)	(0.0844)	(0.0886)

Robust standard errors in parentheses; *** p<0.01, ** p<0.05, * p<0.1. Column (1) uses as a dependent variable the result of principal component analysis comprising all other institutional trust variables. Column (2)-(10) use as dependent variable trust in a specific institution indicated by the column heading. Dependent variables are standardized, coefficients represent outcome's change in terms of standard deviations.

Table A13 Model Table R9 with post-stratification weights

	(1) Chinese Government	(2) US Government	(3) WHO	(4) CDC	(5) Trump	(6) Chinese Public	(7) US Public
Wave 2	-0.0500	-0.0381	-0.0495	-0.196***	-0.113**	-0.221***	-0.0875*
	(0.0403)	(0.0438)	(0.0442)	(0.0629)	(0.0552)	(0.0448)	(0.0518)
Wave 3	-0.346***	0.222***	0.153***	-0.124**	-0.241***	-0.281***	0.442***
	(0.0508)	(0.0559)	(0.0483)	(0.0611)	(0.0505)	(0.0513)	(0.0600)
Wave 2*Trump voter	0.0206	-0.0716	0.00333	0.124	0.286***	0.0772	-0.0397
	(0.0780)	(0.0758)	(0.0719)	(0.0956)	(0.0957)	(0.0768)	(0.0792)
Wave 3*Trump voter	0.299***	-0.179*	-0.0327	0.201*	0.194*	0.120	-0.224**
	(0.0944)	(0.105)	(0.0797)	(0.110)	(0.111)	(0.0966)	(0.0995)
Constant	0.115***	-0.0168	-0.0234	0.0366	0.0617***	0.103***	-0.0898***
	(0.0175)	(0.0185)	(0.0171)	(0.0240)	(0.0218)	(0.0184)	(0.0203)
Observations	2,281	2,281	2,278	2,281	2,281	2,280	2,280
R-squared	0.057	0.033	0.028	0.018	0.031	0.051	0.110
Participants	973	973	972	973	973	973	973
Test Wave 3 – Wave 2=0	-0.296***	0.260***	0.203***	0.0714	-0.128**	-0.0601	0.530***
	(0.0536)	(0.0596)	(0.0500)	(0.0586)	(0.0522)	(0.0499)	(0.0622)
Trump (Wave 3 – Wave 2)*Trump=0	0.278***	-0.107	-0.0360	0.0773	-0.0918	0.0427	-0.184*
	(0.0918)	(0.101)	(0.0862)	(0.107)	(0.107)	(0.102)	(0.0963)

Robust standard errors in parentheses; *** p<0.01, ** p<0.05, * p<0.1. Dependent variables represent to what extent respondents attribute responsibility for the current COVID-19 situation in the US to the actor indicated in the column heading. Dependent variables are standardized, coefficients represent outcome's change in terms of standard deviations.

Table A14 Model Table 3 with post-stratification weights

	(1) Trust in The Federal Government	(2)	(3) Social Trust	(4)
Wave 2	-0.0471	-0.0370	0.0242	0.0149
	(0.0354)	(0.0344)	(0.0260)	(0.0239)
Wave 3	-0.162***	-0.118***	0.00437	-0.0405
	(0.0500)	(0.0399)	(0.0419)	(0.0316)
Direct COVID-19 Exposure		-0.00160		0.0560
		(0.0800)		(0.0700)
Direct COVID-19 Exposure*Trump voter		-0.0753		0.196*
		(0.138)		(0.115)
Cumulative COVID-19 Deaths	0.0663**		-0.0171	
	(0.0257)		(0.0247)	
Cumulative COVID-19 Deaths*Trump voter	-0.119**		-0.00893	
	(0.0475)		(0.0378)	
Constant	-0.0435**	-0.0482***	-0.0790***	-0.0889***
	(0.0209)	(0.0179)	(0.0168)	(0.0145)
Observations	2,272	2,283	2,272	2,283
R-squared	0.020	0.013	0.003	0.013
Participants	966	973	966	973
Test Wave 3 – Wave 2=0	-0.115**	-0.0811**	-0.0198	-0.0554*
	(0.0447)	(0.0397)	(0.0357)	(0.0323)

Robust standard errors in parentheses; *** p<0.01, ** p<0.05, * p<0.1. Columns (1)-(2) use trust in the Federal Government, as a dependent variable, while columns (3)-(4) use a pca variable of social trust, comprising three items for the evaluation of generalized social trust. Dependent variables are standardized, coefficients represent outcome's change in terms of standard deviations.

Table A15 Model Table 4 with post-stratification weights

	(1)	(2)	(3)	(4)
	Baseline		Interaction Trump Voter dummy	
	Social Trust	Trust in the Federal Government	Social Trust	Trust in the Federal Government
Positive Priming	-0.000674	-0.00408	-0.0364	0.0138
	(0.0421)	(0.0607)	(0.0475)	(0.0716)
Negative Priming	-0.0149	-0.0711	-0.0970*	0.0263
	(0.0430)	(0.0645)	(0.0557)	(0.0772)
Trump Voter			0.0420	0.293***
			(0.0663)	(0.0991)
Positive Priming*Trump Voter			0.0142	-0.119
			(0.102)	(0.136)
Negative Priming*Trump Voter			0.161*	-0.311**
			(0.0945)	(0.148)
Cumulative COVID-19 Deaths	-0.00494	0.0279	-0.0120	0.0339
	(0.0152)	(0.0269)	(0.0151)	(0.0275)
Direct COVID-19 Exposure	-0.0186	-0.0384	-0.0178	-0.0434
	(0.0361)	(0.0621)	(0.0368)	(0.0639)
Took part in previous waves	-0.0122	-0.392	-0.115	-1.257***
	(0.123)	(0.537)	(0.134)	(0.163)
Social Trust (pre-experiment)	0.875***		0.868***	
	(0.0160)		(0.0174)	
Political Trust (pre-experiment)		0.636***		0.602***
		(0.0298)		(0.0342)
Constant	-0.0348	-0.0739	0.190	1.093**
	(0.288)	(0.747)	(0.306)	(0.533)
Observations	964	965	909	910
R-squared	0.796	0.483	0.793	0.484
Test Negative Priming – Positive Priming=0	-0.0142	-0.0670	-0.0606	0.0124
	(0.0405)	(0.0637)	(0.0491)	(0.0777)
Test(Negative Priming – Positive Priming)*Trump=0			0.146	-0.192
			(0.104)	(0.146)

Robust standard errors in parentheses; *** p<0.01, ** p<0.05, * p<0.1. The dependent variable in column (1) and (3) represent the extent respondents believe that "most people can be trusted" vs "you cannot be too careful". The dependent variable in column (2) and (4) represent how much respondents think that the Federal Government can be trusted to do what is right. Dependent variables are standardized, coefficients represent outcome's change in terms of standard deviations. Regressions control for socio-demographic characteristics including ethnicity, employment status, education, and their income. Additional controls include indirect (the number of deaths per-capita, at the time of the response, in the panelist's county) and direct COVID-19 exposure, i.e. whether the respondent or one of their loved ones got a COVID-19 diagnosis, a panel dummy indicating if the respondent was part of the panel. Finally, a pre-experimental measure of the dependent value is added. For social trust it is a pca variable including the three items asked in the panel. For political trust, it is the result of pca on trust in Trump, the Federal Government, and Local Governments.

Table A16 Model Table A17 with post-stratification weights

	(1)	(2)	(3)	(4)
	High Social Media Exposure		Low Social Media Exposure	
	Social Trust	Trust in the Federal Government	Social Trust	Trust in the Federal Government
Positive Priming	0.0533	-0.0601	-0.0804	0.0666
	(0.0669)	(0.120)	(0.0713)	(0.0929)
Negative Priming	-0.0541	0.0720	-0.0881	0.0170
	(0.0785)	(0.132)	(0.0761)	(0.0959)
Trump Voter	0.149*	0.446**	-0.00228	0.137
	(0.0842)	(0.184)	(0.0993)	(0.122)
Positive Priming*Trump Voter	-0.0223	-0.0460	0.0221	-0.159
	(0.144)	(0.238)	(0.142)	(0.174)
Negative Priming*Trump Voter	0.239*	-0.484**	0.0246	-0.140
	(0.124)	(0.234)	(0.131)	(0.189)
Cumulative COVID-19 Deaths	-0.0101	-0.0426	-0.00462	0.0993**
	(0.0216)	(0.0460)	(0.0225)	(0.0415)
Direct COVID-19 Exposure	-0.0877	-0.000224	0.00626	-0.0637
	(0.0551)	(0.102)	(0.0524)	(0.0803)
Took part in previous waves	0.0625	-1.051***	Omitted	Omitted
	(0.155)	(0.238)		
Social Trust (pre-experiment)	0.869***		0.855***	
	(0.0242)		(0.0277)	
Institutional Trust (pre-experiment)		0.532***		0.677***
		(0.0520)		(0.0457)
Constant	0.224	-0.246	-0.163	0.454
	(0.495)	(0.875)	(0.389)	(0.461)
Observations	433	434	476	476
R-squared	0.826	0.486	0.787	0.506
Test Negative Priming – Positive Priming=0	-0.107	0.132	-0.00768	-0.0496
	(0.0696)	(0.116)	(0.0675)	(0.108)
Test (Negative Priming – Positive Priming)*Trump=0	0.262*	-0.438*	0.00254	0.0195
	(0.154)	(0.225)	(0.131)	(0.187)

Robust standard errors in parentheses; *** p<0.01, ** p<0.05, * p<0.1. The sample in column (1) and (2) are individuals who reported a social media exposure score above the median, column (3) and (4) are based on individuals with social media exposure below the median. The dependent variable in column (1) and (3) represent the extent respondents believe that "most people can be trusted" vs "you cannot be too careful". The dependent variable in column (2) and (4) represent how much respondents think that the Federal Government can be trusted to do what is right. Dependent variables are standardized, coefficients represent outcome's change in terms of standard deviations. Regressions control for socio-demographic characteristics including ethnicity, employment status, education, and their income. Additional controls include indirect (the number of deaths per-capita, at the time of the response, in the panelist's county) and direct COVID-19 exposure, i.e. whether the respondent or one of their loved ones got a COVID-19 diagnosis, a panel dummy indicating if the respondent was part of the panel. Finally, a pre-experimental measure of the dependent value is added. For social trust it is a pca variable including the three items asked in the panel. For political trust, it is the result of pca on trust in Trump, the Federal Government, and Local Governments.

Table A17 Model Table 4 (column (3)-(4)) split by social media exposure

	(1)	(2)	(3)	(4)
	High Social Media Exposure		Low Social Media Exposure	
	Social Trust	Trust in the Federal Government	Social Trust	Trust in the Federal Government
Positive Priming	0.0408	0.0320	-0.111*	0.0915
	(0.0675)	(0.103)	(0.0612)	(0.0884)
Negative Priming	-0.0603	-0.0523	-0.109	0.0716
	(0.0748)	(0.125)	(0.0688)	(0.0876)
Trump Voter	0.156**	0.550***	-0.0161	0.113
	(0.0766)	(0.172)	(0.0823)	(0.115)
Positive Priming*Trump Voter	-0.0633	-0.281	0.0396	-0.153
	(0.123)	(0.221)	(0.117)	(0.160)
Negative Priming*Trump Voter	0.194*	-0.425*	0.0222	-0.137
	(0.109)	(0.218)	(0.116)	(0.169)
Cumulative COVID-19 Deaths	-0.0272	-0.0252	0.000139	0.0714*
	(0.0239)	(0.0431)	(0.0209)	(0.0394)
Direct COVID-19 Exposure	-0.0257	-0.0751	0.0140	0.0198
	(0.0492)	(0.0872)	(0.0489)	(0.0703)
Took part in previous waves	-0.0145	-1.199***	Omitted	Omitted
	(0.156)	(0.216)		
Social Trust (pre-experiment)	0.866***		0.868***	
	(0.0222)		(0.0248)	
Institutional Trust (pre-experiment)		0.502***		0.692***
		(0.0477)		(0.0416)
Constant	0.253	1.249	-0.0783	-0.277
	(0.482)	(0.781)	(0.318)	(0.519)
Observations	433	434	476	476
R-squared	0.806	0.486	0.783	0.477
Test Negative Priming – Positive Priming=0	-0.101	-0.0842	0.00277	-0.0199
	(0.0640)	(0.112)	(0.0607)	(0.0987)
Test (Negative Priming – Positive Priming)*Trump=0	0.257**	-0.144	-0.0175	0.0165
	(0.125)	(0.207)	(0.111)	(0.170)

Robust standard errors in parentheses; *** p<0.01, ** p<0.05, * p<0.1. The sample in column (1) and (2) are individuals who reported a social media exposure score above the median, column (3) and (4) are based on individuals with social media exposure below the median. The dependent variable in column (1) and (3) represents the extent respondents believe that "most people can be trusted" vs "you cannot be too careful". The dependent variable in column (2) and (4) represents how much respondents think that the Federal Government can be trusted to do what is right. Dependent variables are standardized, coefficients represent outcome's change in terms of standard deviations. Regressions control for socio-demographic characteristics including ethnicity, employment status, education, and their income. Additional controls include indirect (the number of deaths per-capita, at the time of the response, in the panelist's county) and direct COVID-19 exposure, i.e. whether the respondent or one of their loved ones got a COVID-19 diagnosis, a panel dummy indicating if the respondent was part of the panel. Finally, a pre-experimental measure of the dependent value is added. For social trust it is a pca variable including the three items asked in the panel. For political trust, it is the result of pca on trust in Trump, the Federal Government, and Local Governments.

Additional Figures

Figure A1 Model Figure 1 with post-stratification weights

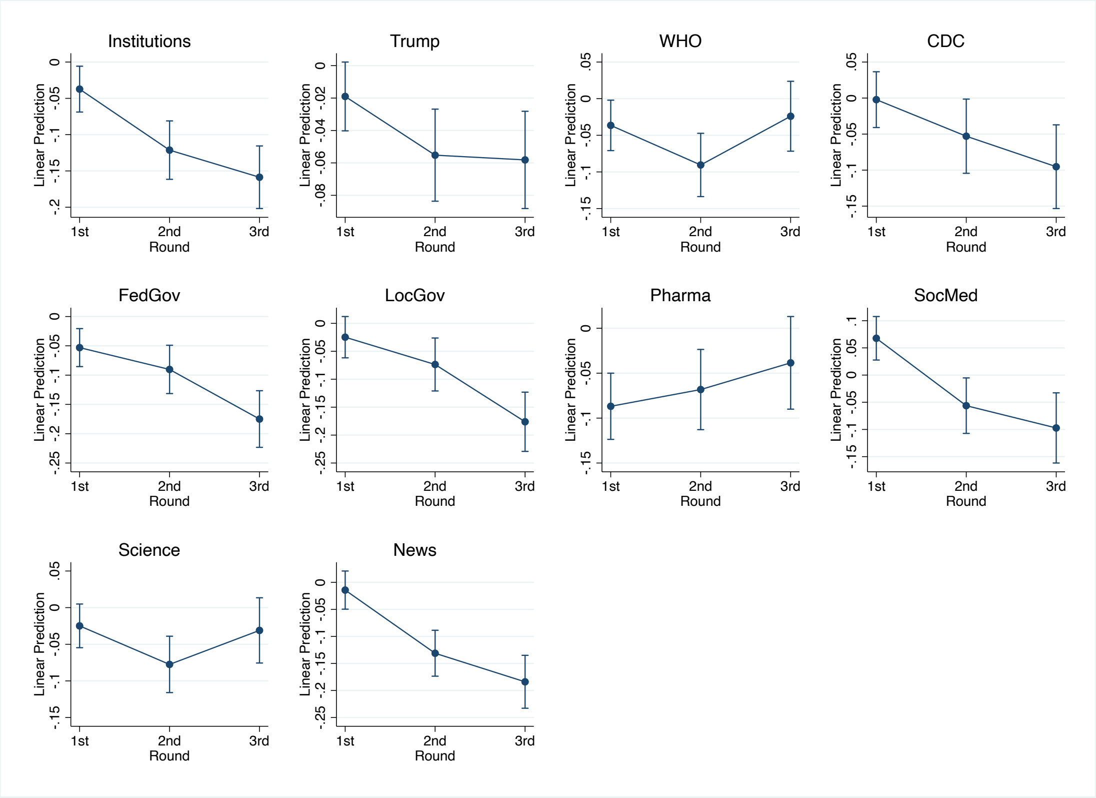

Figure A2 Model Table R2

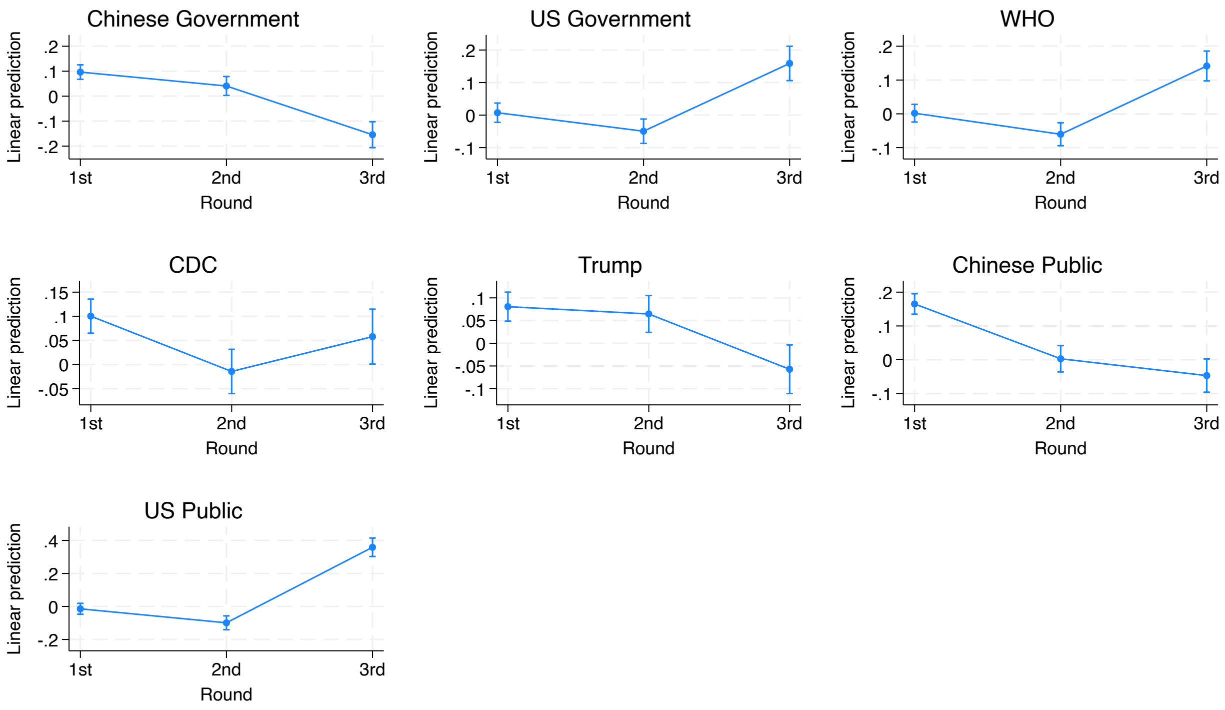

Figure A3 Model Figure A2 with post-stratification weights

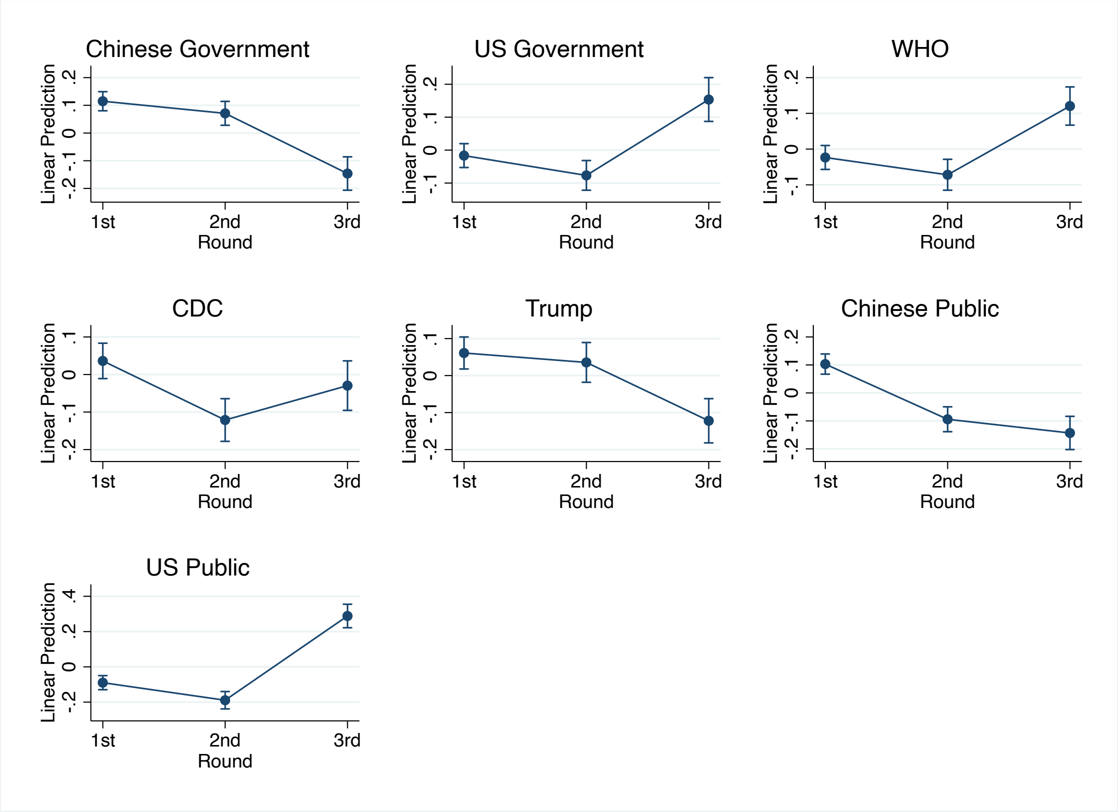

Figure A4 Model Figure 2 with post-stratification weights

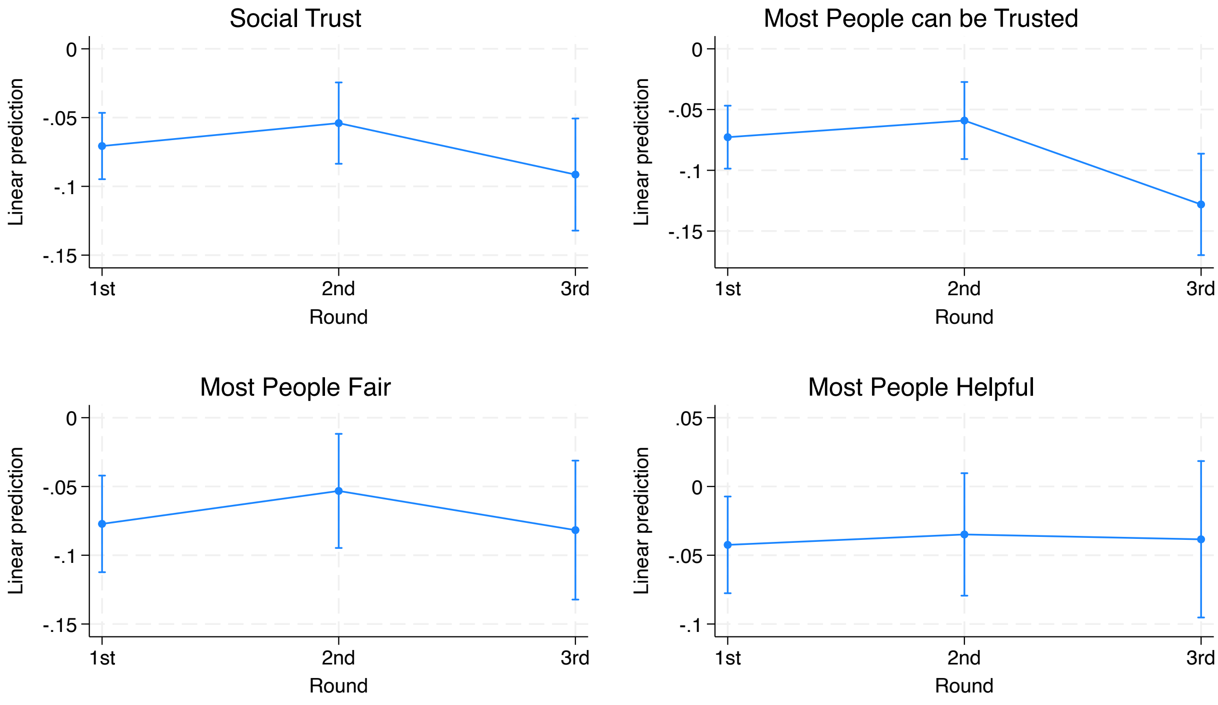

Figure A5 Model Figure 3 with post-stratification weights (Panel A) and alternative version with representation of coefficients (Panel B)

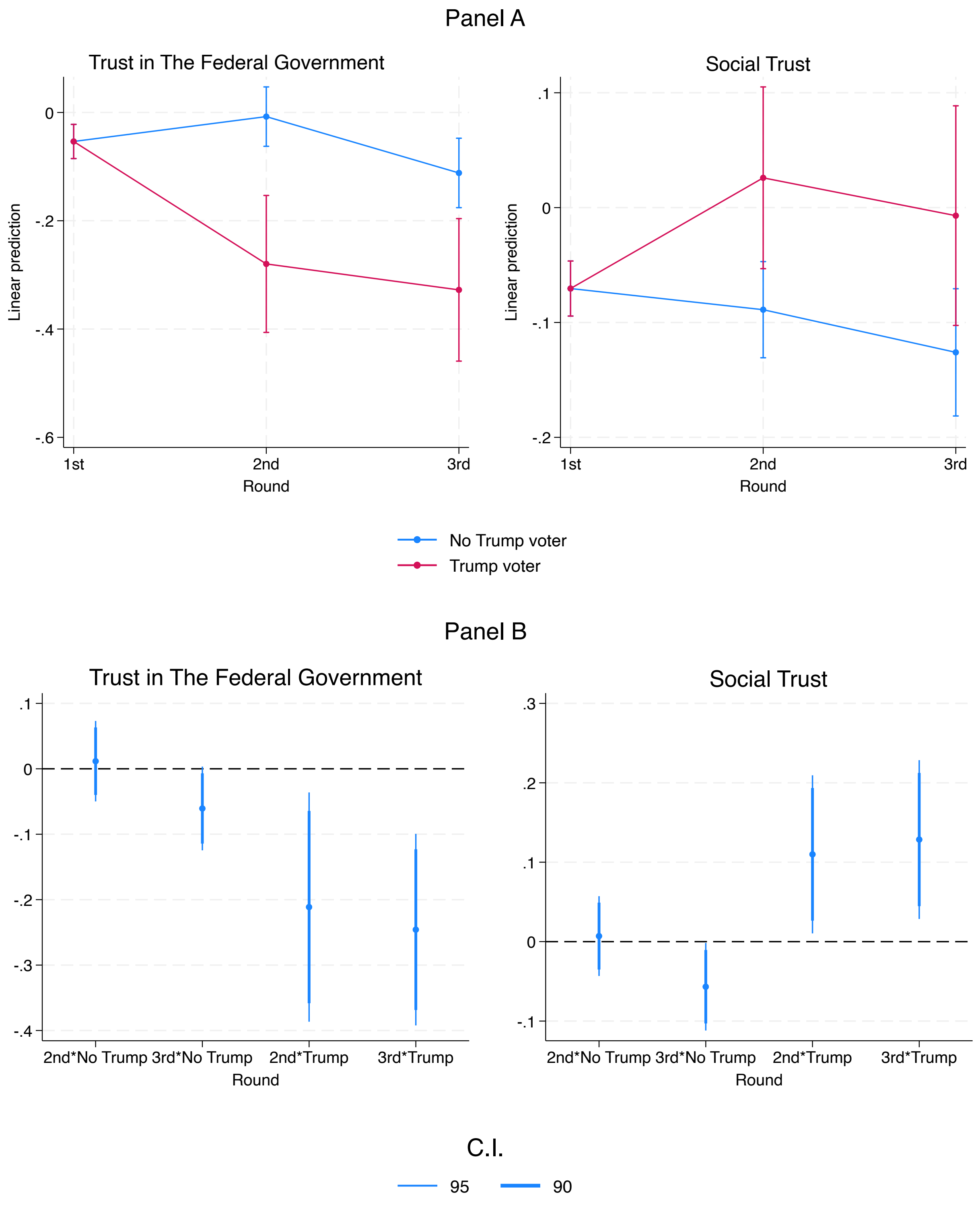

Figure A6 Model Figure 4 with post-stratification weights

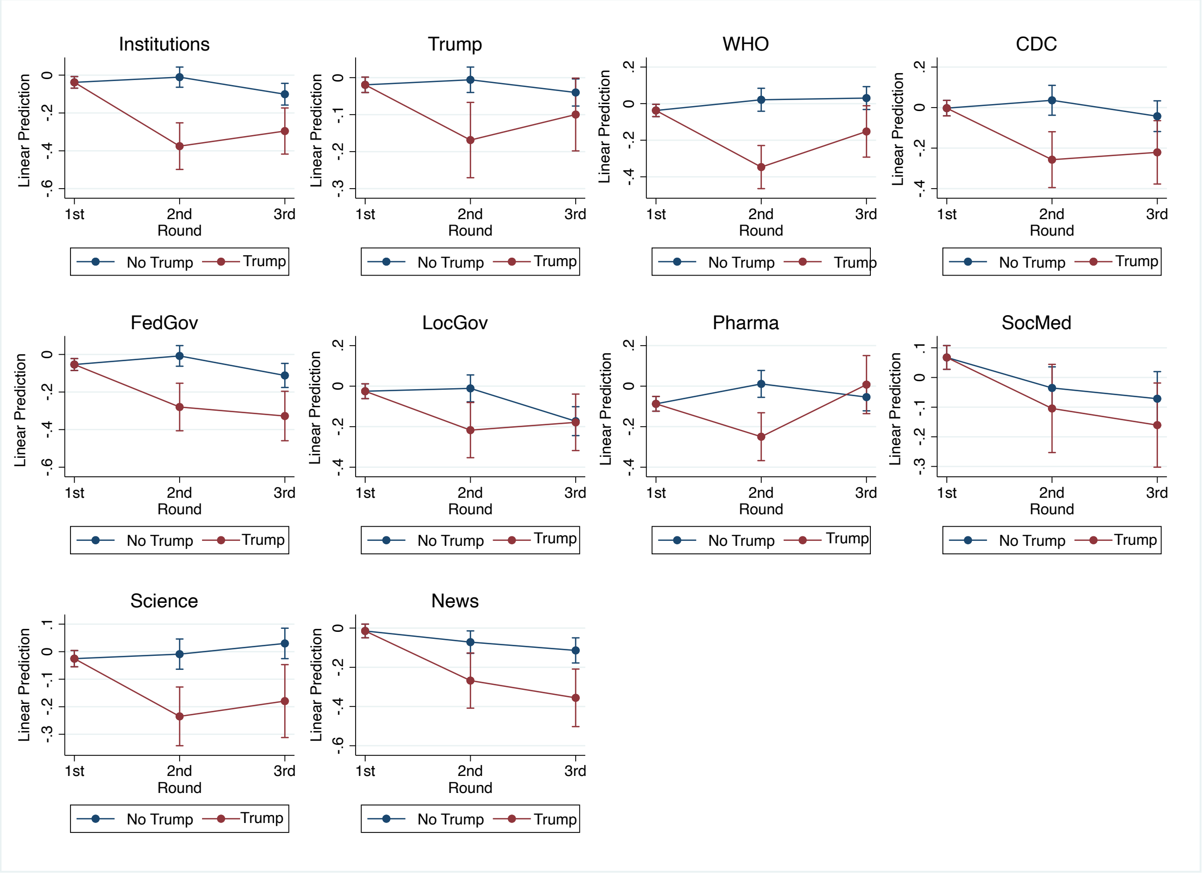

Figure A7 Model Table R9

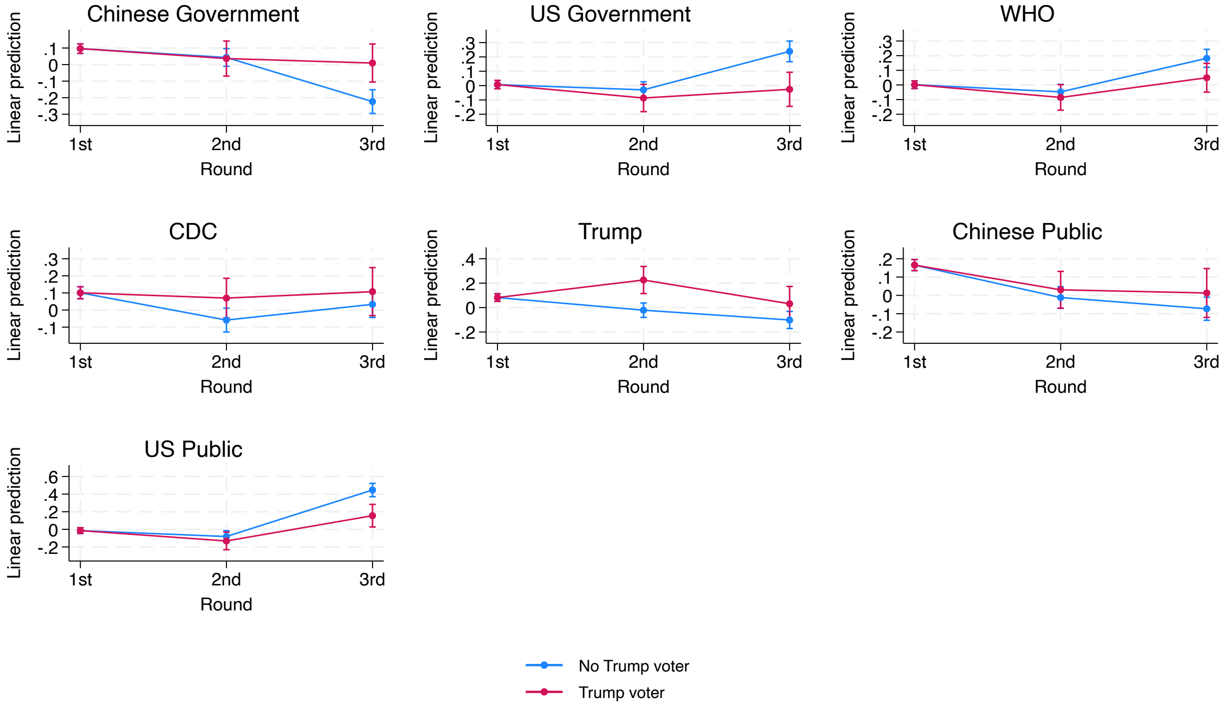

Figure A8 Model Figure A7 with post-stratification weights

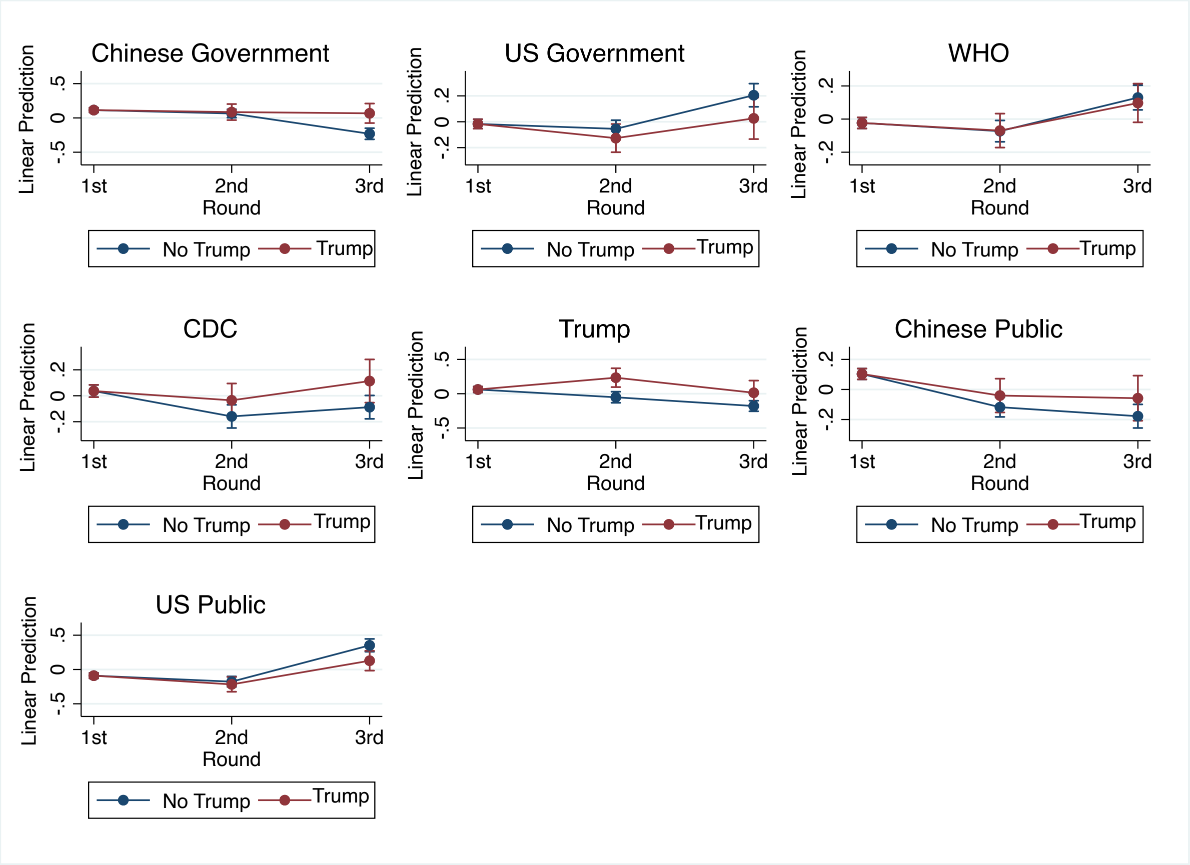

Figure A9. Graphical visualization of coefficients from Table R1

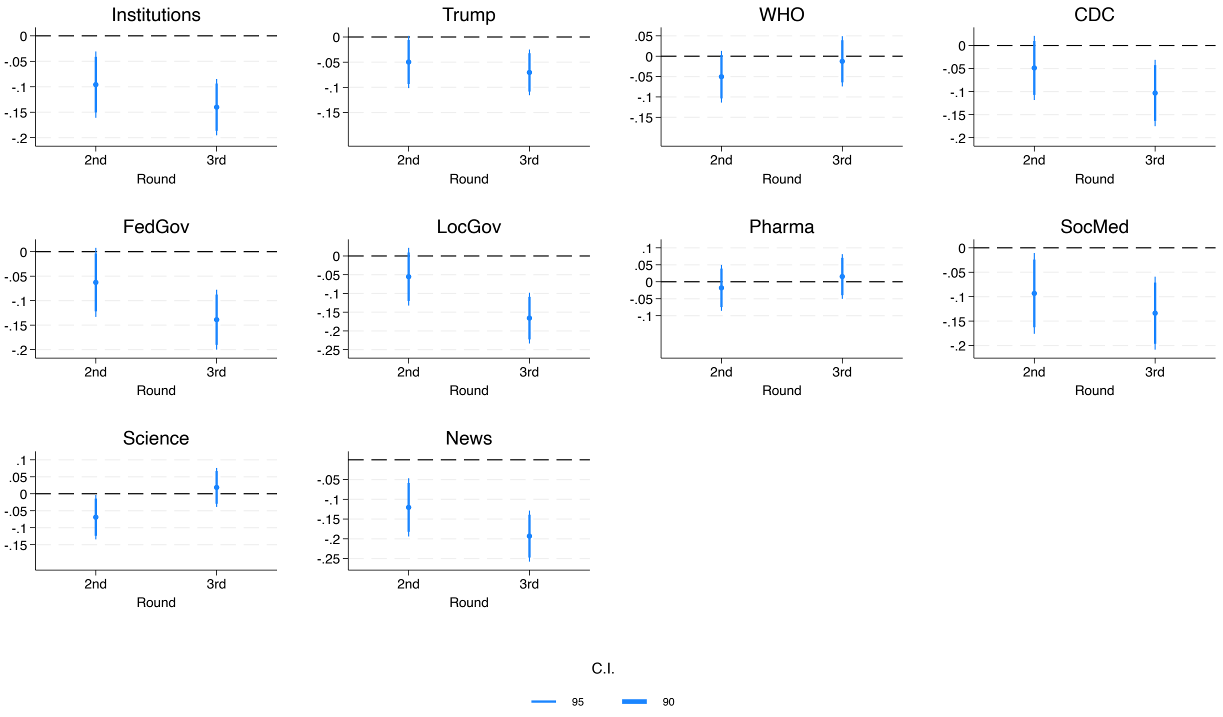

Figure A10. Graphical visualization of coefficients from Table R2

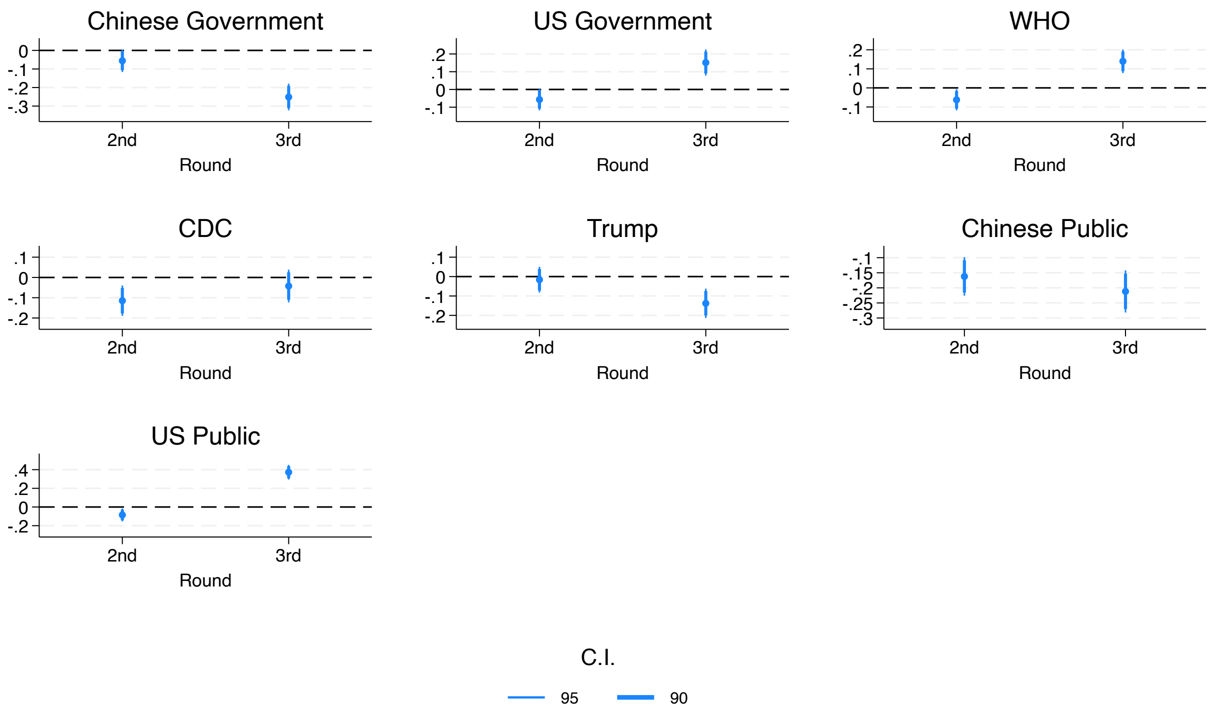

Figure A11. Graphical visualization of coefficients from Table R3

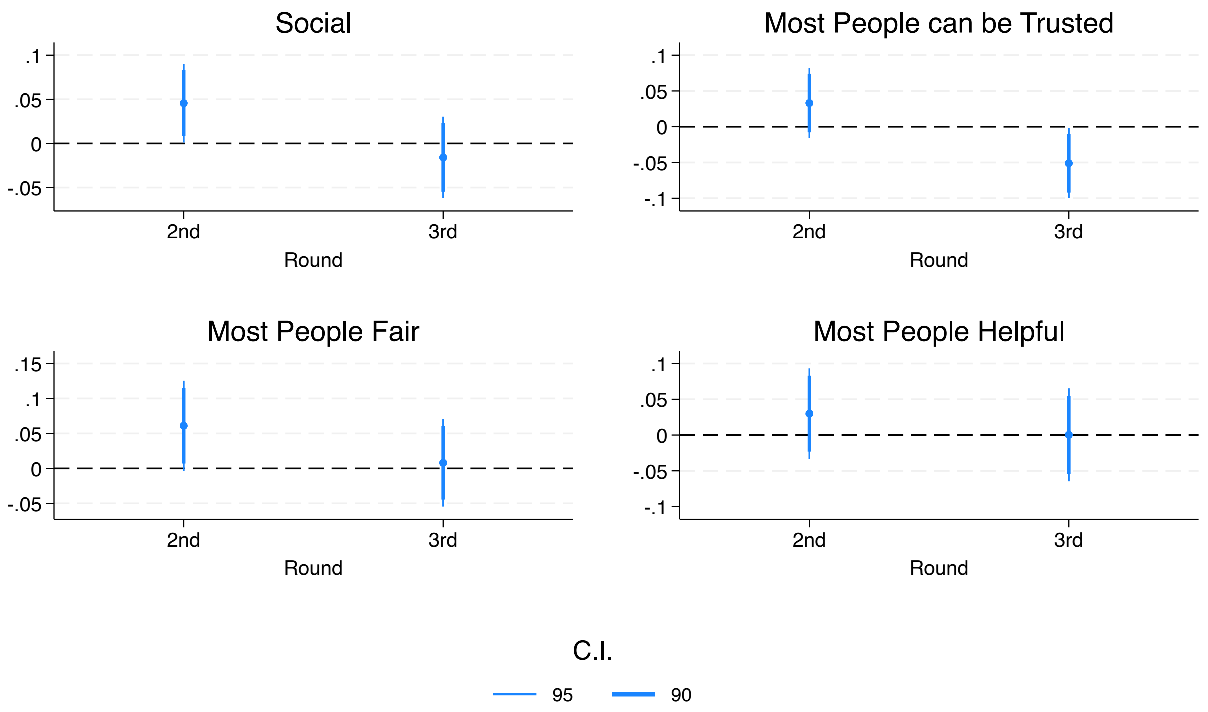

Figure A12. Graphical visualization of coefficients from Table R6

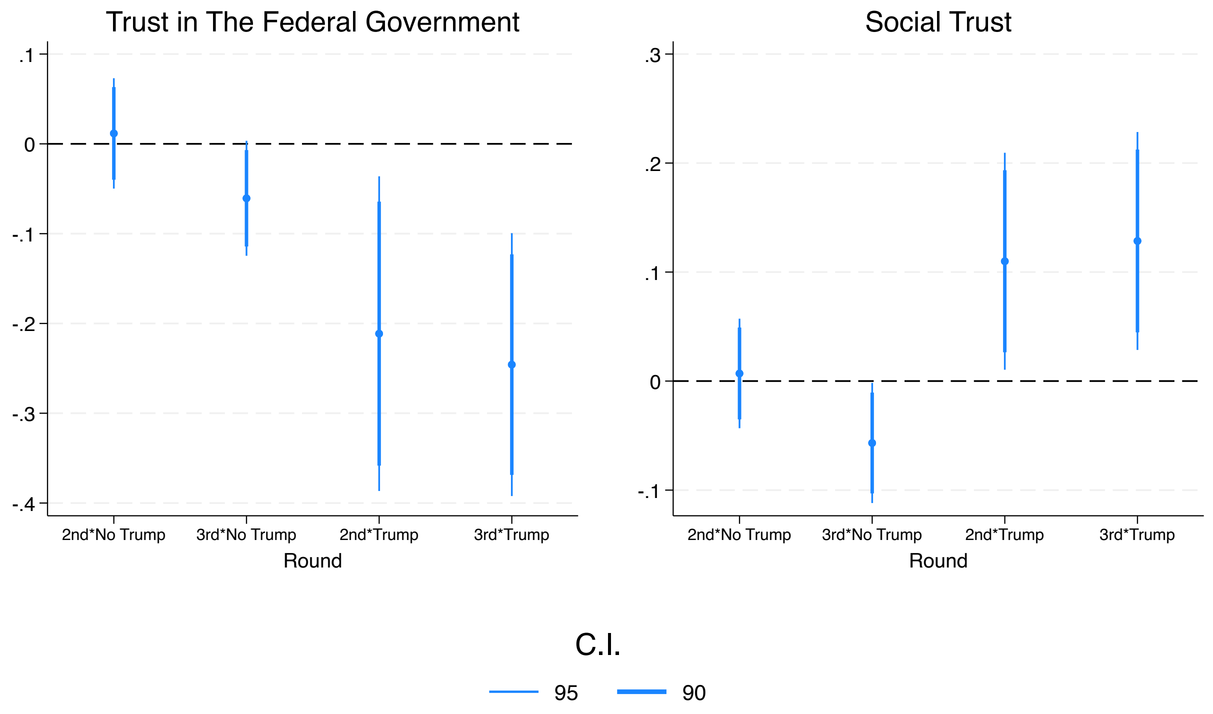

Figure A13. Graphical visualization of coefficients from Table R8

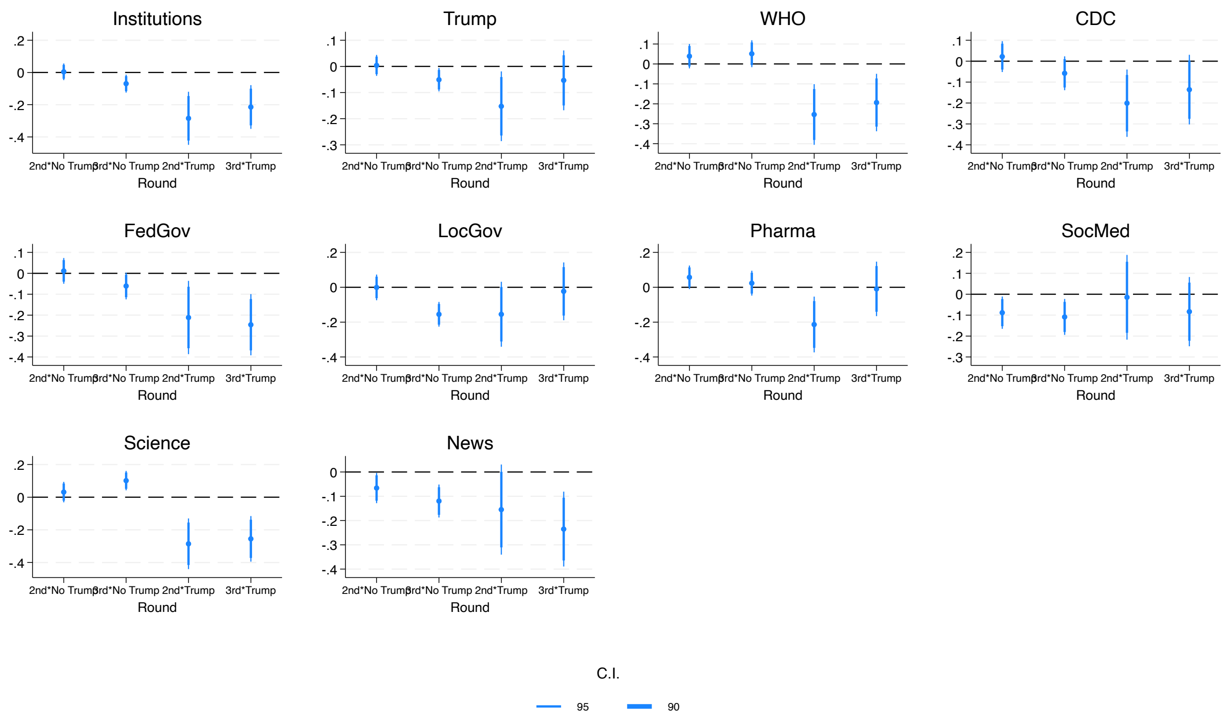

Figure A14. Graphical visualization of coefficients from Table R9

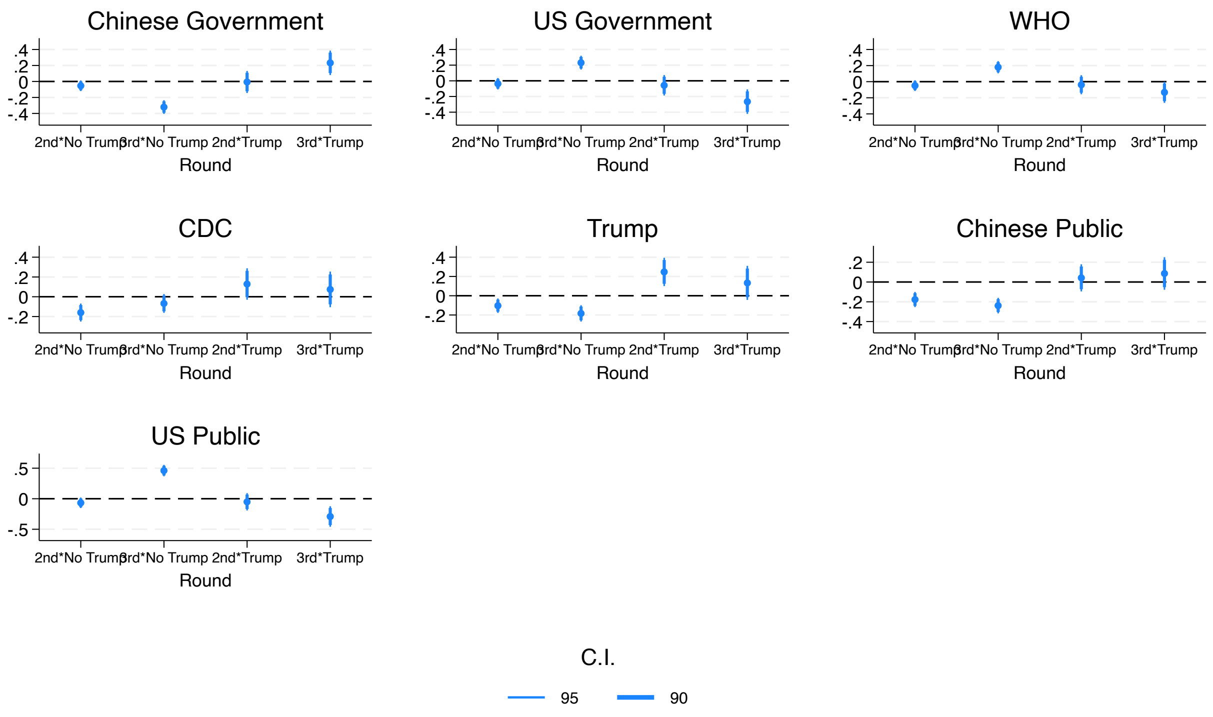

Figure A15. Dynamics of Trust in the Federal Government by unexplained probability of being Trump voter

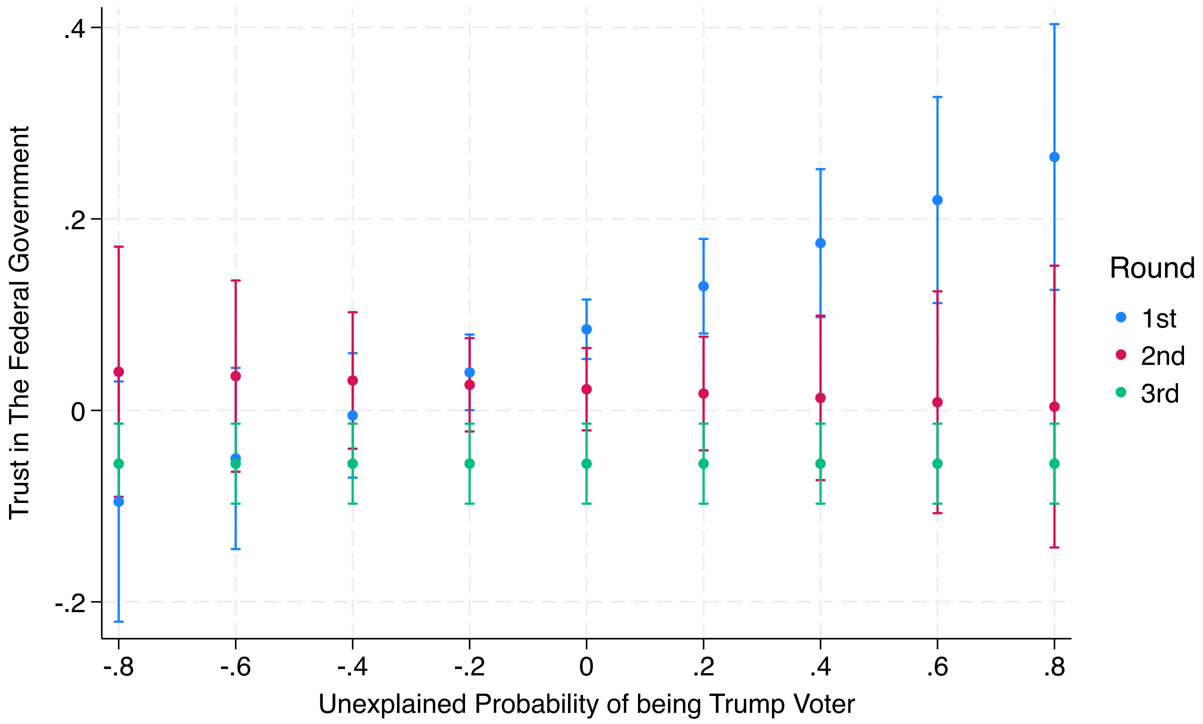

Notes: Margins from the fixed effect regression of trust in the Federal Government on round fixed effects and on the interaction between round dummies and Trump voter residuals (unexplained probability of being Trump voter). Bars represent the marginal effect of round on trust in the Federal Government for the level of unexplained probability of being Trump voter.

Figure A16. Dynamics of Social Trust by unexplained probability of being Trump voter

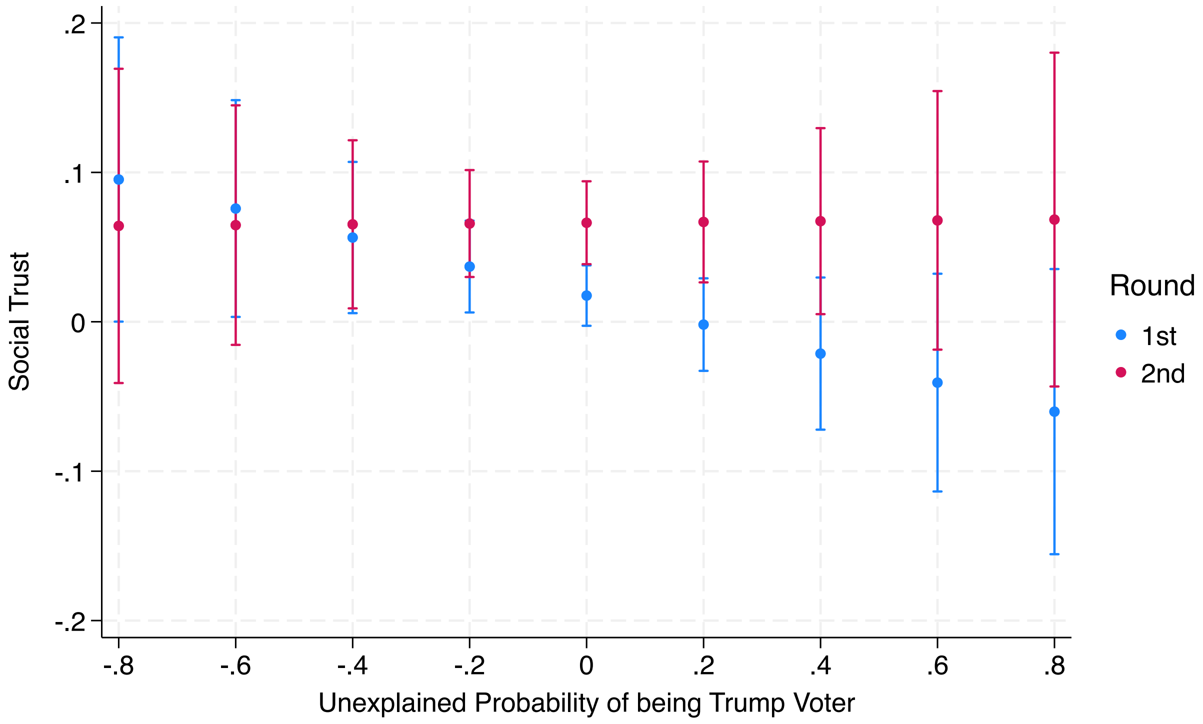

Notes: Margins from the fixed effect regression of social trust on round fixed effects and on the interaction between round dummies and Trump voter residuals (unexplained probability of being Trump voter). Bars represent the marginal effect of round on social trust for the level of unexplained probability of being Trump voter.

Figure A17. Effect of Direct COVID-19 Exposure on Trust in the Federal Government by unexplained probability of being a Trump voter

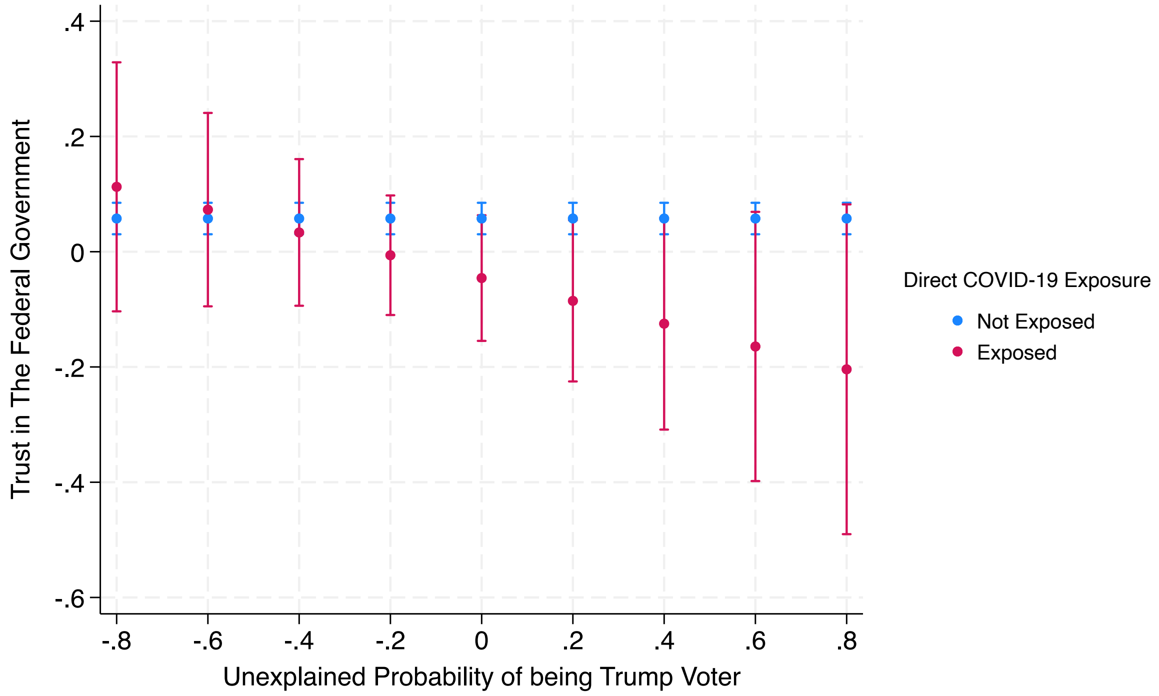

Notes: Margins from the fixed effect regression of trust in the Federal Government on round fixed effects, direct COVID-19 exposure and on the interaction between direct COVID-19 exposure and Trump voter residuals (unexplained probability of being Trump voter). Bars represent the marginal effect of direct COVID-19 exposure on trust in the Federal Government for the level of unexplained probability of being Trump voter.

Figure A18. Effect of Direct COVID-19 Exposure on Social Trust by unexplained probability of being a Trump voter

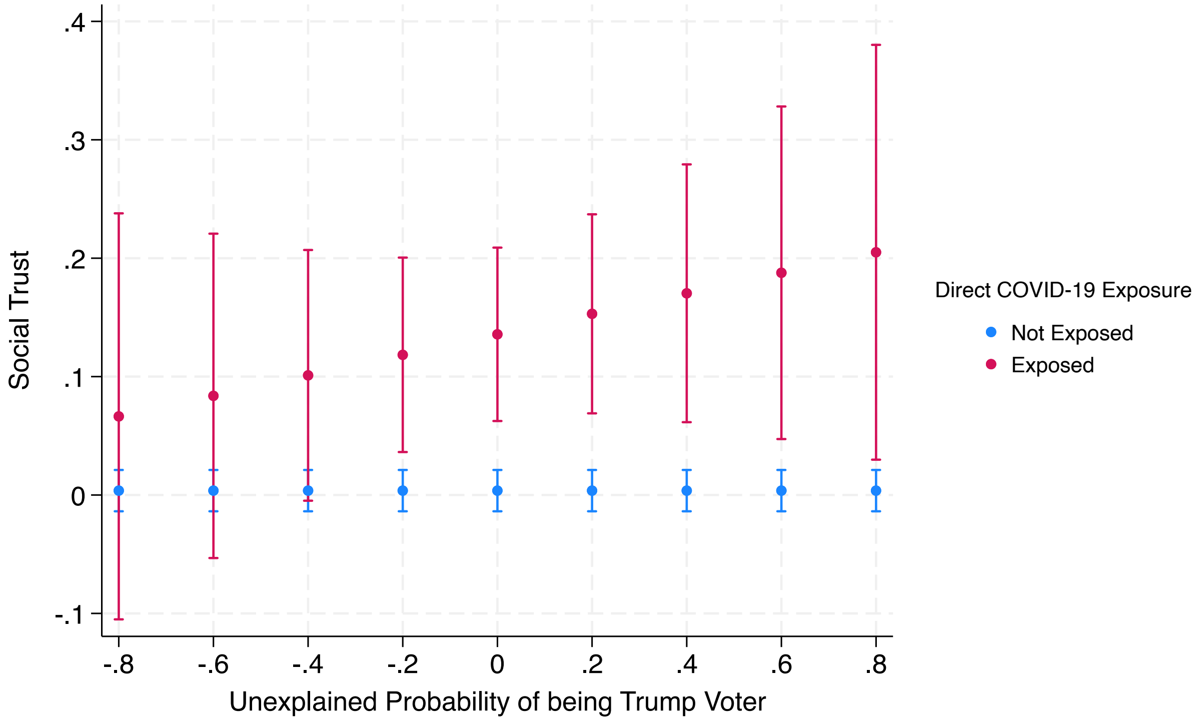

Notes: Margins from the fixed effect regression of social trust on round fixed effects, direct COVID-19 exposure and on the interaction between direct COVID-19 exposure and Trump voter residuals (unexplained probability of being Trump voter). Bars represent the marginal effect of direct COVID-19 exposure on social trust for the level of unexplained probability of being Trump voter.

Figure A19. Impact of priming conditions on the perception of the health and economic situation

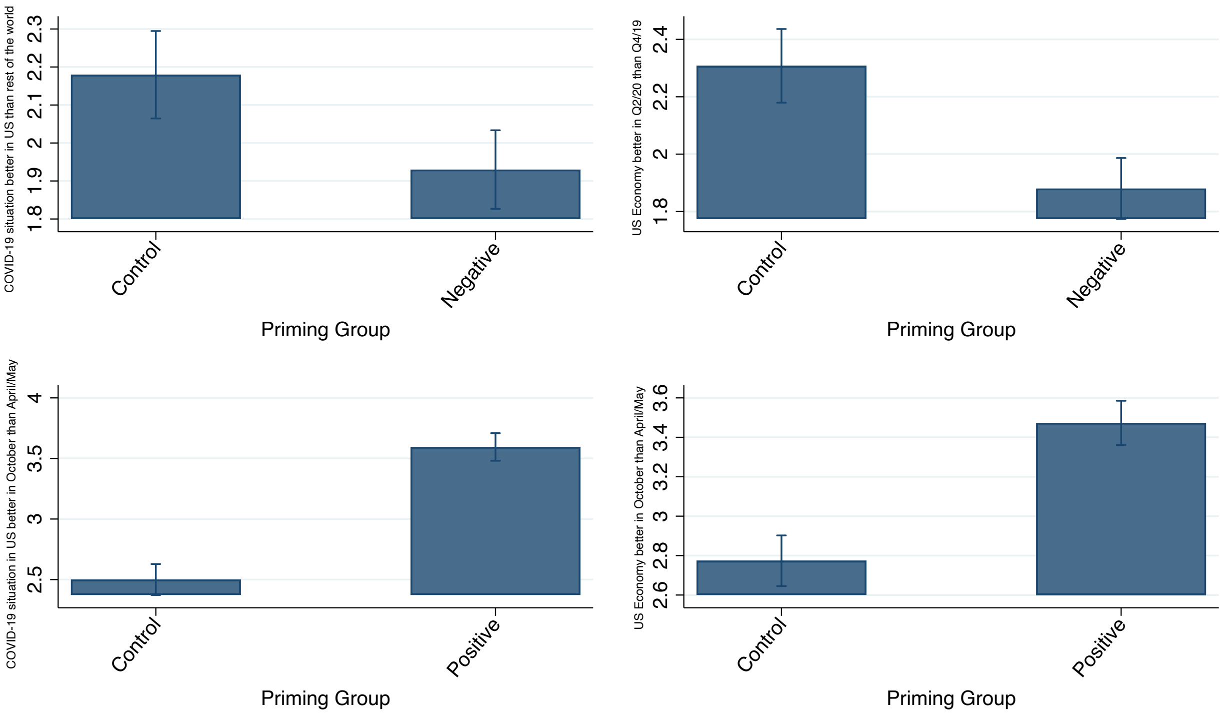

Notes: Margins measure fact perception (“Would you say that the COVID-19 situation in the US is better, about the same, or worse than the COVID-19 situation in the rest of the world?” in the top left; “Would you say that the nation's economy during the second quarter of 2020 has gotten better, stayed about the same, or gotten worse compared to the economy during the last quarter of 2019?” in the top right; “Would you say that the recent COVID-19 situation in the US has improved, stayed about the same, or worsened compared to that in the April/May?” in the bottom left; “Would you say that the nation's economy in more recent times has improved, stayed about the same, or worsened compared to that in the March/April?” in the bottom right), where answers vary from “Very much improved” (5) to “Very much worsened” (1), in the different priming groups. Regressions that generated margins controlled for ethnicity, voting, education, employment, income, participation to the panel, and both direct and indirect COVID-19 exposure. Each question was asked in the control group and one of the treatment groups.

Figure A20. Positive vs Negative message perception of priming

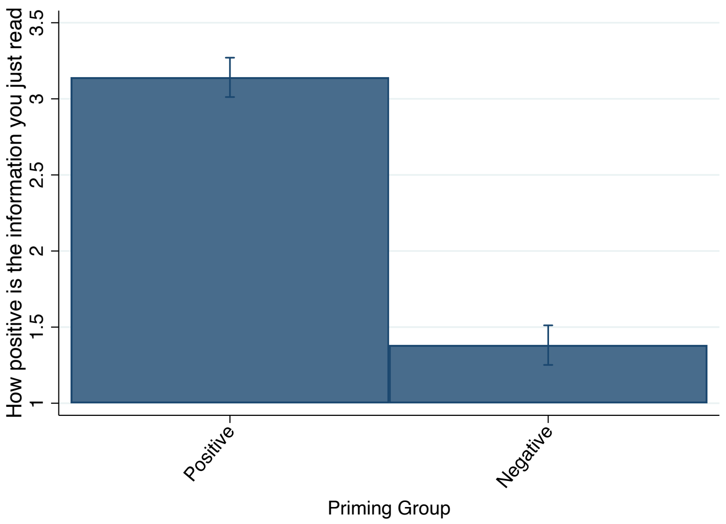

Notes: Margins measure “How positive or negative is the information you just read on describing how the US has been coping with the COVID-19 pandemic?”, where answers are rescaled from “very negative” (0) to “very positive” (5) in the two treatment groups. The regression that generated margins controlled for ethnicity, voting, education, employment, income, participation to the panel, and both direct and indirect COVID-19 exposure.

Figure A21. Partisan effects of priming conditions on the perception of the health and economic situation

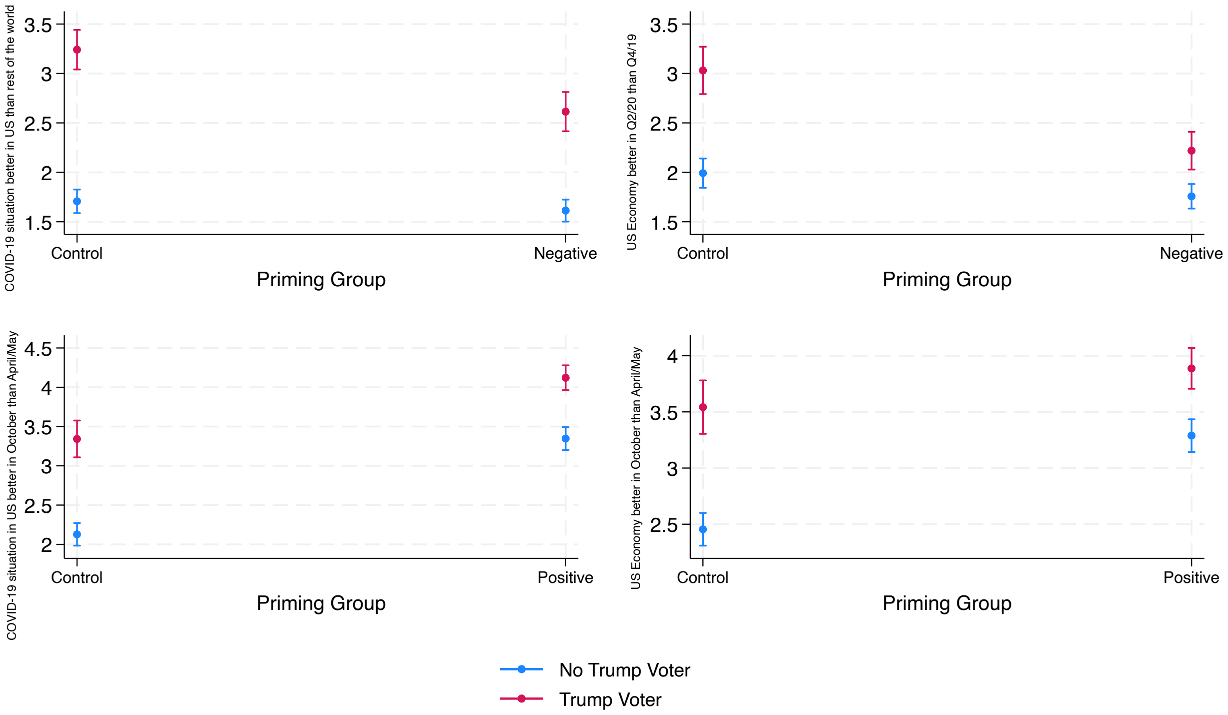

Notes: Margins measure fact perception ("Would you say that the COVID-19 situation in the US is better, about the same, or worse than the COVID-19 situation in the rest of the world?" in the top left; "Would you say that the nation's economy during the second quarter of 2020 has gotten better, stayed about the same, or gotten worse compared to the economy during the last quarter of 2019?" in the top right; "Would you say that the recent COVID-19 situation in the US has improved, stayed about the same, or worsened compared to that in the April/May?" in the bottom left; "Would you say that the nation's economy in more recent times has improved, stayed about the same, or worsened compared to that in the March/April?" in the bottom right), where answers vary from "Very much improved" (5) to "Very much worsened" (1), in the different priming groups for Trump voters and non-Trump voters. Regressions that generated margins controlled for ethnicity, education, employment, income, participation to the panel, and both direct and indirect COVID-19 exposure. Each question was asked in the control group and one of the treatment groups.

Figure A22. Positive vs Negative message perception of priming, by political orientation

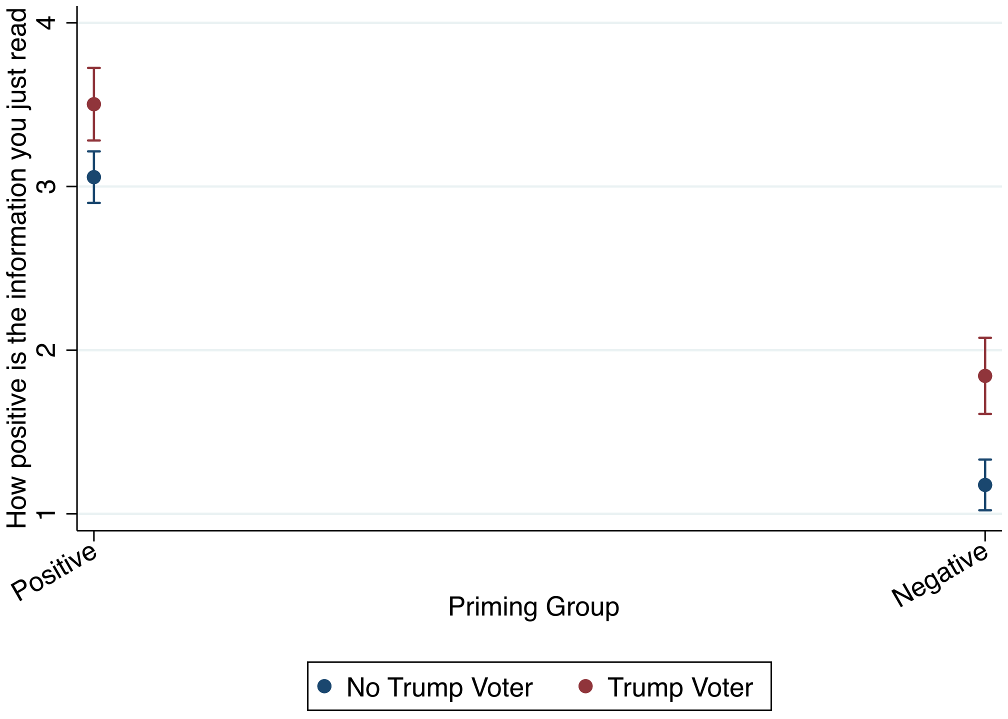

Notes: Margins measure "How positive or negative is the information you just read on describing how the US has been coping with the COVID-19 pandemic?", where answers are rescaled from "very negative" (0) to "very positive" (5) for Trump voters versus non-Trump voters in the two treatment groups. The regression that generated margins controlled for ethnicity, education, employment, income, participation to the panel, and both direct and indirect COVID-19 exposure.
